# Meso/macroscopically multifunctional surface interfaces, ridges, and vortex-modified anode/cathode cuticles as force-driven modulation of high-energy density of LIB electric vehicles

**DOI:** 10.1038/s41598-019-51345-z

**Published:** 2019-10-11

**Authors:** H. Khalifa, S. A. El-Safty, A. Reda, M. A. Shenashen, M. M. Selim, O. Y. Alothman, N. Ohashi

**Affiliations:** 1 National Institute for Materials Science (NIMS), Sengen 1-2-1, Tsukuba, Ibaraki 305-0047 Japan; 2 Department of Mathematics, Al-Aflaj College of Science and Human Studies, Prince Sattam Bin Abdulaziz University, Al-Aflaj, 710-11912 Saudi Arabia; 3 Chemical Engineering Department, College of Engineering, King Saud University, P.O. Box 800, Riyadh, 11421 Saudi Arabia; 4 Research Center for Functional Materials, National Institute for Materials Science, 1-1 Namiki, Tsukuba, Ibaraki 305-0044 Japan

**Keywords:** Energy, Batteries, Batteries

## Abstract

Modulation of lithium-ion battery (LIB) anodes/cathodes with three-dimensional (3D) topographical hierarchy ridges, surface interfaces, and vortices promotes the power tendency of LIBs in terms of high-energy density and power density. Large-scale meso-geodesics offer a diverse range of spatial LIB models along the geodetically shaped downward/upward curvature, leading to open-ended movement gate options, and diffusible space orientations. Along with the primary 3D super-scalable hierarchy, the formation of structural features of building block egress/ingress, curvature cargo-like sphere vehicles, irregularly located serrated cuticles with abundant V-undulated rigidness, feathery tube pipe conifers, and a band of dagger-shaped needle sticks on anode/cathode electrode surfaces provides high performance LIB modules. The geodetically-shaped anode/cathode design enables the uniqueness of all LIB module configurations in terms of powerful lithium ion (Li^+^) movement revolving in out-/in- and up-/downward diffusion regimes and in hovering electron density for high-speed discharge rates. The stability of built-in anode//cathode full-scale LIB-model meso-geodesics affords an outstanding long-term cycling performance. The full-cell LIB meso-geodesics offered 91.5% retention of the first discharge capacity of 165.8 mAhg^−1^ after 2000 cycles, Coulombic efficiency of ~99.6% at the rate of 1 C and room temperature, and high specific energy density of ≈119 Wh kg^−1^. This LIB meso-geodesic module configuration may align perfectly with the requirements of the energy density limit mandatory for long-term EV driving range and the scale-up commercial manufactures.

## Introduction

In the last decade, extensive studies have been devoted develop the geometric and characteristic features of rechargeable LIBs as clean environmentally and sustainable energy^1^. To date, LIBs are widely used as power sources for modern portable electronic devices, such as smart grids, laptops, electronic gadgets, camcorders cameras, and smart phones^1,2^. LIBs with high energy density would become imperative requirements in future renewable energy storage systems. The expanding utilization of LIBs for zero-emission transportation applications, such as plug-in hybrid (PHEVs) and fully electric vehicles (EVs), have been recognized commercially due to their extreme values of volumetric and gravimetric energy and power densities^3–9^. A minimum driving mileage of ∼300 miles indicated the energy density of 100 Whkg^−1^. This characteristic value is typically considered the threshold to ensure the prosperity of next-generation EVs^10^. The rapid growth in the development of EV manufacture in the automotive market remains a challenge due to the cost-to-range proportion. Therefore, remarkable efforts to develop novel fabrication routes of sustainable anode/cathode materials and inexpensive and safe designs of LIB battery pack systems are highly needed.

To control the geometric LIB electrode designs, hybrid materials based on LiMPO_4_/olivine isostructures (where M is a transition metal, such as Mn, Fe, Co, or Ni) were introduced as basis for the next-generation Li^+^-ion secondary battery cathodes^3,11,12^. Among these structures, lithium iron phosphate (LiFePO_4_; LFPO) compounds with a regular olefin structure have assumed the leading materials to be used as efficient electrode fabrication due to their multiple features, such as good structural stability, high thermal safety, abundance of raw materials, intrinsic safety, low toxicity, eco-friendliness, high specific capacity, excellent cycle stability, and cost effectiveness^12–15^. LFPO materials showed a plateau around the electrode voltage of 3.5 V versus Li/Li^+^ with a theoretical discharge capacity of 170 mAhg^−1^ ^16,17^. However, several obstacles and difficulties curbed the extensive implementation of LFPO cathodes in zero-emission transfer applications, such as components in hybrid components (PHEVs) and fully EVs. These limitations include poor electronic and ionic conductivities, unsatisfactory rate capability, sluggish kinetics of LFPO-electrode and lithium-ion transport, and low tap density^11,18–21^. Therefore, several efforts have been devoted to overcome these disadvantages and limitations and improve the electron/ion transportation reversible capacity and excellent rate capability of LFPO-based electrode. In this regard, attempts through control of (i) hierarchical structure design, (ii) reduction of the particle size, and (iii) synthesis of three-dimensional (3D) structure orientations would improve multi-diffusivity, and open sites for Li^+^ ion transports^19^. Typical strategies that are successful for improvements the LFPO surface interfaces and functionalities were by doping other metals or carbon and coating the carbon-film layers around the LFPO grains^19,22–27^. If the electrode materials are fabricated into 3D topographical meso-geodesics that have surfaces with hierarchy ridges, downward/upward curvatures, interfaces, their diverse applications in the configuration of spatial energy storage models can be widely expanded^28,29^.

Furthermore, nanoscale, transition metal oxides became promising anode materials for high performance LIBs^30^. Among all metal oxides, titanium dioxide (TiO_2_) with anatase phase structure is suggested as an excellent anode candidate owing to its great merits in safety performance, low cost, nontoxicity, pollution-free nature, eco-friendliness, low polarization, and good cyclic stability and reversibility^25,31,32^. The anodic TiO_2_ merits could be due to the structural transition from tetragonal TiO_2_ (space group I4_1_/amd) into orthorhombic lithium-rich Li_0.5_TiO_2_ (space group Imma)^33^. In addition, the recombination of electrons and holes observed along the band gap of TiO_2_ and its composites may afford actively functional surfaces of anodic TiO_2_^34^. To improve the performance of LIBs, efforts have been devoted to develop simple, yet efficient, design strategies of specific electrode materials to facilitate high transfer rates of Li^+^ and diminish length diffusion. The performance of LIBs depends mainly on the charge/discharge levels associated with electron/Li^+^-ion migration through the electrode and electrolyte. In this regard, various carbon nanostructures and their composites have been widely applied for electrode fabrication in electrochemical energy storage gadgets due to their remarkable carbon chemical stability^35–38^. Therefore, taking advantage of the hybrid/doped nanostructure of carbon materials and metal oxides bears importance in improving TiO_2_-based electrode materials in LIBs.

The modulation of the anode/cathode nanomaterial composites in final fabrication of highly variable, non-prescribed and geometric LIB models remains a challenge. To achieve impressive LIB models, the anode/cathode structural stability to withstand formidable life-use cycles is crucial. The structural collapse tendency of electrodes under charge/discharge cycles has diminished their functions in LIB pack modules. Herein, periodically meso-geodesic anode/cathode electrode models were designated to afford stable LIB modules with high levels of heavy Li^+^ truck loads, non-resisting spread electrons/Li^+^ transports, and potential occupant diffusions after multiple discharge (insertion, lithiation)/charge (delithiation, extraction) cycles. The synergetic contributions of two-force anode/cathode meso-geodesic structures that fabricated with multifunctional surface interfaces included irregular ripples, bumps, and undulations and anticlines are key components in configuration of LIB modules. A series of mesogeodesic LFPO artichoke flower hollows (AF@C) that feature convex spheres and serrated cuticles, conjugated hollow spheres (CHS@C), hairy coconut spheres (HCS@C) as cathode, and TiO_2_@C porous nano-sink hole-like vortex (PHV@C) anode hierarchy was fabricated. Results showed that the full-scale built-in integrations of AF@C cathode// PHV@C anode LIBs provide an outstanding long-term cycling performance with 91.5% capacity retention at a first discharge capacity of 165.8 mAhg^−1^ and excellent Coulombic efficiency of ~99.6% after 2000 cycles in the potential region from 0.8 V to 3.5 V versus Li/Li^+^ at the rate of 1 C and at room temperature. The proposed full-scale LIB meso-geodesic models offer a high value of specific energy density of ≈119 Wh kg^−1^. The overall finding indicates the structural effect of meso-geodesics on the uniqueness of all module configurations. The geodetically-shaped anode/cathode design enables retention of multidiffusive systems, electron/ion flow gradients, and channel gates after multiple cycles. This full-scale LIB meso-geodesic module with outstanding energy density, safety characteristics, and cycling durability is promising for the requirements needed to improve the driving range of EVs and the scale-up commercial manufactures.

## Experimental

### Synthesis of 3D topographical meso-geodesic hierarchy cathodes

Under hydrothermal treatment, anion-assisted synthesis can control the fabrication of heterogeneous structurally shaped spheroids along the hierarchy artichoke-flower full-open-geode model (AF), hairy coconut sphere (HCS@C), and conjugated hollow sphere (CHS) cathode. In typical synthesis of the AF structure, the elemental molar ratio of Li:Fe:P in the spheroids of the formulated and main composition domains of iron(III) nitrate nonahydrate, phosphoric acid, and Li salts was equivalent to 3:1:1. Each sample solution [i.e., phosphoric acid or iron(III) nitrate nonahydrate] was first prepared by dissolving each compound in a mixture of 5 mL Milli-Q water, 2.5 mL ethanol, and 3000 µL ethylene glycol and then stirring separately for 1 h. After stirring the two sample solutions, phosphoric acid solution was added dropwise (0.5 mL/min) to the iron(III) nitrate nonahydrate solution under continuous stirring for another 1 h. Finally, a solution of LiCl dissolved in 10 mL Milli-Q water, 5 mL ethanol, and 6000 µL ethylene glycol was stirred for 1 h at 30 °C. The LiCl solution was added dropwise to the iron(III) nitrate nonahydrate/phosphoric acid mixture at the rate of 0.5 mL/min. The total molar ratio of Li:Fe:P was equivalent to 3:1:1 in the mixture. The final mixture was treated under vigorous magnetic stirring for another 6 h at pH of 7. The final AF mixtures were transferred into 100 mL Teflon-lined stainless-steel autoclaves. The autoclaves were maintained at 170 °C for 12 h and cooled to room temperature. The resulting solid AF products were centrifuged, washed thrice with Milli-Q water and absolute ethanol, and dried overnight at 60 °C under vacuum. The final powder product was calcined at 600 °C for 6 h to form the AF composite hierarchy. Using the same procedures at pH 7, and molar ratio of Li:Fe:P (i.e., 3:1:1), where different Li sources such as lithium fluoride and lithium carbonate were employed to fabricate HCS and CHS meso-geodesics (Supplementary Information S1–S9).

### Synthesis of anatase TiO_2_ hierarchy with multi-hole-like vortices (PHV anode)

A spherical TiO_2_ anatase structure with massive PHV can be fabricated according to the typical synthesis method. In brief, 1 mL titanium ethoxide was dissolved in a mixture of 20 mL Milli-Q water and HCl (2 mol/L) under gentle stirring for 6 h to form a homogenous solution. About 3 mL of 30% H_2_O_2_ solution was added dropwise at the rate of 0.5 mL/min under vigorous magnetic stirring; stirring was continued for another 2 h. The final mixture was transferred into 100 mL Teflon-lined stainless-steel autoclaves. The autoclaves were maintained at 170 °C for 12 h and cooled to room temperature. The resulting solid products were centrifuged, washed thrice with Milli-Q water and absolute ethanol, and dried overnight at 60 °C under vacuum. The final powder product was calcined at 600 °C for 3 h to form anatase TiO_2_ meso/macro multihole sphere (PHV) anode material (Supplementary Information S1–S9).

### Fabrication of heterogeneous meso-geodesics cathode/anode composites

The multifunctional surface interfaces of cathode/anode composites could be achieved by carbon-shell coating of geodesic LFPO AF with convex spheres and serrated cuticles, CHS, and HCS as cathode, and TiO_2_ (PHV) anode hierarchy under ultrasonication for 15 min. The samples were dispersed in a mixture of glucose/ethanol (5 wt%). Then, the mixtures were transferred to the autoclaves and constantly stirred for another 30 min under microwave irradiation at 80 °C and for 0.5 h. The color of the mixtures gradually turned black. The precipitates were collected using a centrifuge machine and washed thrice with Milli-Q water and ethanol. All precipitates were dried overnight at 55 °C in an electric oven. The resulting samples were milled separately in a mortar and calcined in Ar atmosphere at 350 °C for 0.5 h and then set at 600 °C for 2 h at a heating rate of 5 °C/min. The final products were labeled as PHV@C (anode) and AF@C and HCS@C and CHS@C (cathodes).

### Fabrication of 3D super-scalable models of half-cell and full-scale anode//cathode LIB meso-geodesics

To control the 3D super-scalable LIB model, we designate the LIB meso-geodesics in the CR2032 coin cells for half-and-built-in full-scale LIB models. The built-in anode//cathode full-scale LIB-model meso-geodesics were designated as reported in the patterning steps in Supplementary Information S1. The spatial rate performance capabilities, long-term cycling performance and stability, capacity retention, and average Coulombic efficiency were measured under specific protocols (see for instance Supplementary Information S10–S11).

We fabricated a wide range of 3D super-scalable AF@C//PHV@C cathode//anode LIB meso-geodesics (i.e., a full-scale LIB-model), and cathode half-scale geodesic LFPO@C structures included AF@C, CHS@C and HCS@C LIBs, respectively. In addition, the anode half-scale TiO_2_@C (PHV@C) LIBs are also designated. In all half- and full-scale LIB configurations, we used the liquid electrolyte solution of LiPF_6_ (1 M) conductive salt in ethylene carbonate/diethyl carbonate (1:1 v/v). The working electrodes were prepared by mixing each active meso-geodesic materials of PHV@C anode and AF@C, CHS@C and HCS@C cathodes with carbon black. The carbon black/ meso-geodesic mixture was dissolved in N-methyl-2-pyrrolidone under stirring for 1 h, and then polyvinylidene fluoride was added as a binder at a weight ratio of 75:15:10, respectively. The full-scale cell was fabricated under optimized mass loading control to optimize the full-scale cell (i.e., balancing N/P ratio or negative and positive (N/P) electrode ratio). The full-scale cathode//anode stacked-layer pouch LIB model was applied to determine the areal capacity, and volumetric energy density (see Supplementary Information S12, S13). The mass loadings were 11.85 and 6.49 mg/cm^2^ for the positive cathode and negative anode active materials, respectively. The areal discharge capacities were 1.13 and 1.19 Ah/cm^2^ for the cathode and anode, respectively. The general balancing is based on the equal discharge specific capacity (in Ah) for the N/P electrodes, giving the N/P capacity ratio ((N:P)_Cap_) = 1.05:1. To achieve the optimal trade–off between improvement and safety, oversized N electrode capacity and (N:P)_Cap_ > 1:1 were demanded. In addition, the optimum specific energy mainly indicated the equal capacities of N/P electrodes, that is, the (N:P)_Cap_ = 1:1, for our proposed stacked-layer LiFePO_4_//TiO_2_ pouch LIB-model.

The slurries were spread over aluminum foil (10 µm thickness) for fabrication of LFPO@C cathodes. In turn, the copper foil (8 µm thickness) was used as a platform for fabrication of PHV@C anode. Both cathode/anode film electrodes were dried in a vacuum oven at 80 °C for 12 h. The dried thick-film electrodes were pressed between twin rollers to enhance its packing density, reduce film porosity, and ensure intimate-contact of the active meso-geodesic materials and the current collector. In all half- and full-scale LIB configurations, we used a microporous polymer separator of Celgard 2400TM membrane that supplied from Hoechst Celanese Corporation, Charlotte, North Carolina, USA (see Supplementary Information S1).

To fabricate LIB CR2032 coin cells, circular electrodes with diameters of 16 mm for both Li foil and working electrodes, in addition to a 20 mm-diameter of a circular separator, were punched for further use. A high-quality crimping process was applied by using crimper machine for the CR20XX series coin cells to press the fabricated 2032 coin cells inside the glove box under Ar gas. The designated full-cell AF@C cathode//PHV@C anode LIBs were used for the electrochemical measurements (see Fig. S1). Moreover, full-scale LFPO@C cathode //TiO_2_@C anode stacked-layer pouch LIB models were designed in this work (Supplementary Information S1). To guarantee the total intake of electrolyte solution by the both electrodes, the prepared built-in 3D super-scalable models of half- and full-scale cathode//anode LIB meso-geodesics were kept for 24 h inside the glove box under Ar gas, and prior to electrochemical measurements.

## Results and Discussion

**Figure 1:**
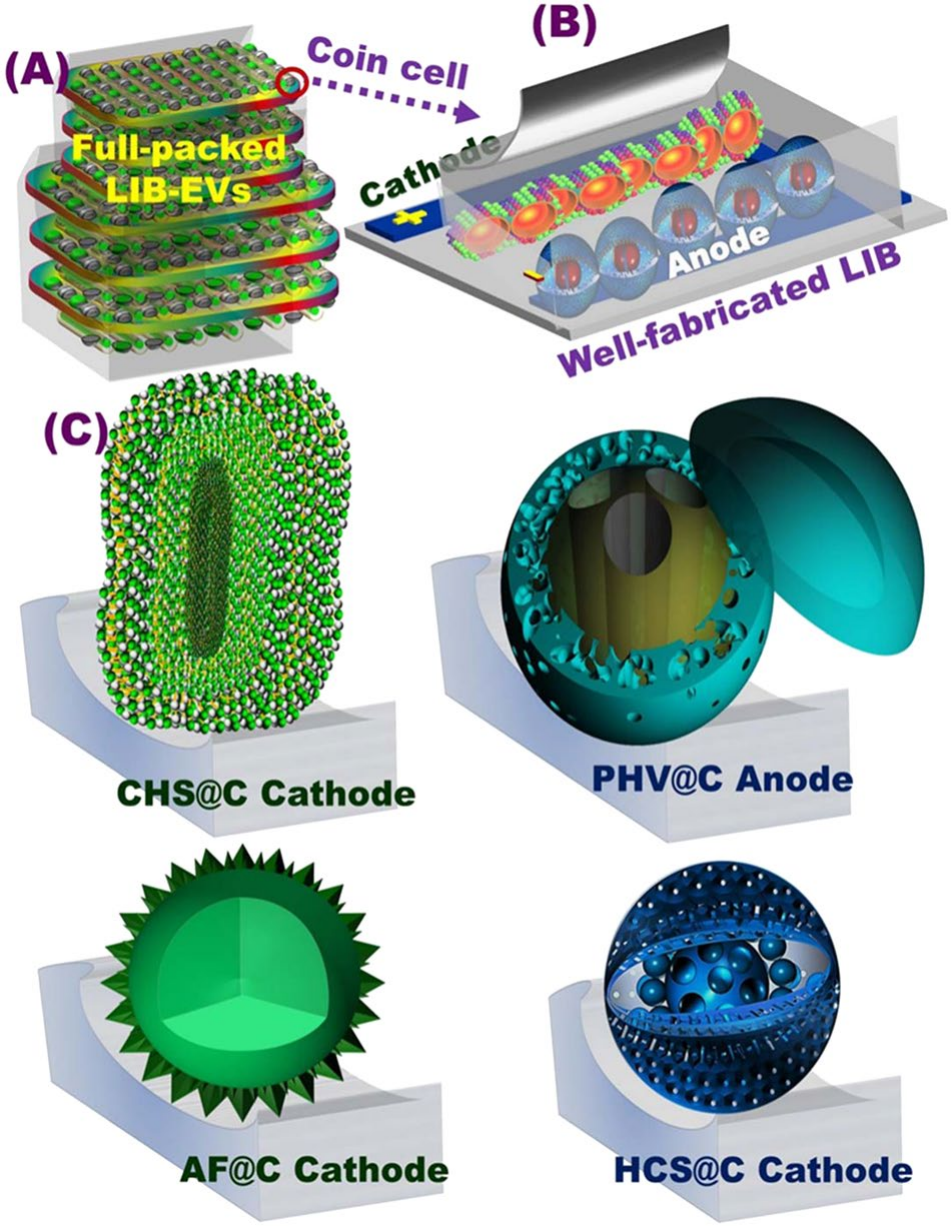
(**A**) Full-packed 18650 cylindrical cells of AF@C//PHV@C full-scale coin cells for creating rechargeable LIBs for EVs. The EV pack was formed via the connection of several built-in modules in a series of LIB 2032 coin cells. The module was designed by connecting a number of LIB coin cells characterized by similar voltages and capacities, to a certain extent, in (i) series (S) and (ii) parallel (P) directions; (**B**) formulation and well-defined fabrication of 3D orientation of meso-geodesic anode/cathode cuticles; 3D topographical hierarchy ridges, surface interfaces, and vortices designated in CR2032 coin cells influencing the power tendency of LIBs in terms of high energy density and power density; (**C**) 3D projections of structurally shaped LFPO hollow spheroid hierarchy (cathode) and anatase TiO_2_@C of multi-hole spheres (PHV@C, anode) represented according to the FE-SEM and HRTEM microscopic patterns of AF@C with hollow-geode cavity and needle dendrites along the 3D anisotropic hollow surfaces, HCS@C, and CHS@C cathodes, in addition to the integrated anatase TiO_2_@C spheres decorated with PHV@C anode in full-scale LIBs.

A control engineering of anode/cathode meso-geodesic hollowness structures that fabricated with multifunctional surface interfaces included irregular ripples, bumps, and undulations and anticlines is key components in unique configuration of LIB modules (see Fig. 1 and Supplementary Information S1–S9). A diverse range of geodetically-shaped cathode/anode models included geodesic LFPO@C sink spaces of AF@C, CHS@C, and HCS@C as cathodes, and TiO_2_@C (PHV@C) anode hierarchy was fabricated. As the primary half- and full-cell LIBs designated with positive LFPO@C-cathode// negative PHV@C anode, the structural building blocks of both cathode/anode surfaces with egress/ingress channels, downward/upward curvature cargo-like sphere vehicles, irregularly located serrated cuticles, abundant V-undulated rigidness, feathery tube pipe conifers, and a band of dagger-shaped needle sticks offer open-ended electron/Li^+^-ion movement options, and retention of diffusion orientations during charge/discharge cycles. Together, our two-force cathode/anode building-electrode designs would entirely modulate meso-geodesics with multifunctional electron/Li^+^-ion gateways along surface interfaces, vortices, curvature angularities, spherule cortices, cave blocks, and undulated rigidness, leading to build substantial and high performance LIB models (Supplementary Information S1–S11).

Figure 1A,B shows simple design of 3D super-scalable, full-packed LIB CR2032 coin cells. The schematic projections suggested the full-scale LFPO@C cathode //TiO_2_@C anode stacked-layer pouch LIB models, as shown in the patterning steps. Figure 1B shows the topographic objects and morphological surface electrode meso-geodesics, which associated with multi-diffusible meso/macro-windows, inter-trapped cage cavity spaces, and multi-vast-mouth caves in the entire hollow nests. In general, the geodetically-shaped meso-architectures with their surface functions and vortices enabled (i) facile movements of Li^+^ ions along in/out directions, and (ii) fast and high loading capacity of anode and cathode electrodes (Fig. 1C). Our designated 3D super-scalable models of full-scale anode//cathode LIB CR2032 coin cells are significantly effective in improving the spatial rate performance capabilities, long-term cycling performance and stability, capacity retention, and average Coulombic efficiency compared with the common-scale LIBs (Fig. 1A,B).

**Figure 2:**
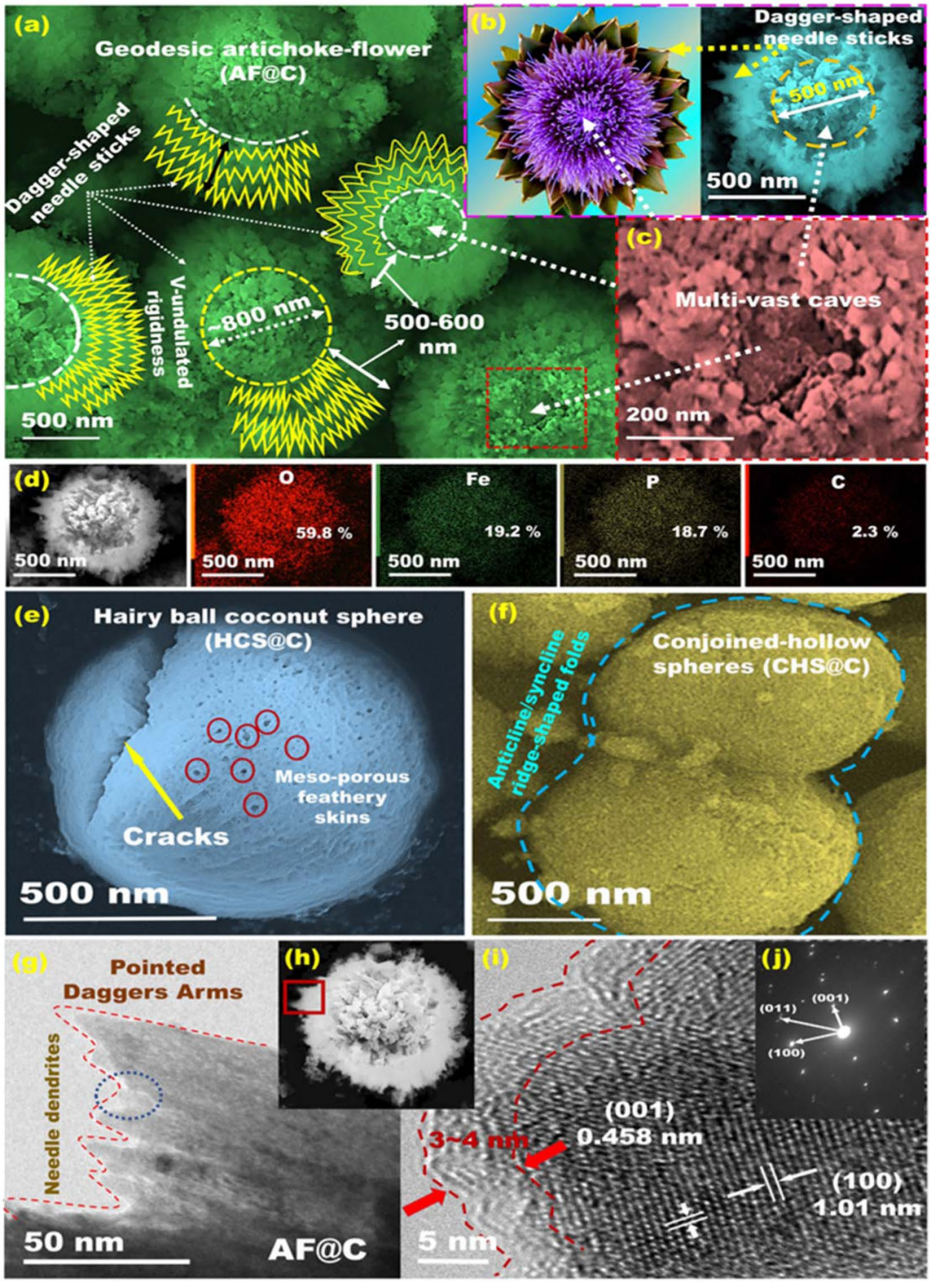
(**a**–**j**) FE-SEM, EDX–elemental mapping analysis, HR-TEM, and electron diffraction profiles of the exposed heterogeneous hierarchy open-geode AF@C model, HCS@C, and CHS@C cathode composites. (**a**,**b**) Low- and (**d**) high-magnification FE-SEM of LFPO@C with geodesic AF@C (cathode) with serrated cuticle walls and channel-like needles running along the exterior feathery tube pipes. (**d**) EDX and elemental mapping analysis (mass ratios) of AF@C geode of O (59.8%), Fe (19.2%), P (18.7%), and C (2.3%). (**e**), and (**f**) represented the FE-SEM of HCS@C, and CHS@C, respectively. (**g**) High- and (**h**) low-magnification HR-TEM micrographs at the edge of hand-like pointed daggers on the surface of AF@C microparticle. (**i**) High-magnification HR-TEM of polyhedron lattice pattern at the edge with a clear thin layer (3–4 nm) of C coating on the surface of AF crystals. (**j**) SAED pattern image of AF@C meso-geodesics along the [010] crystallographic direction indicating the formation of a single orthorhombic olivine mesocrystals with the AF@C meso-geodesics.

**Figure 3:**
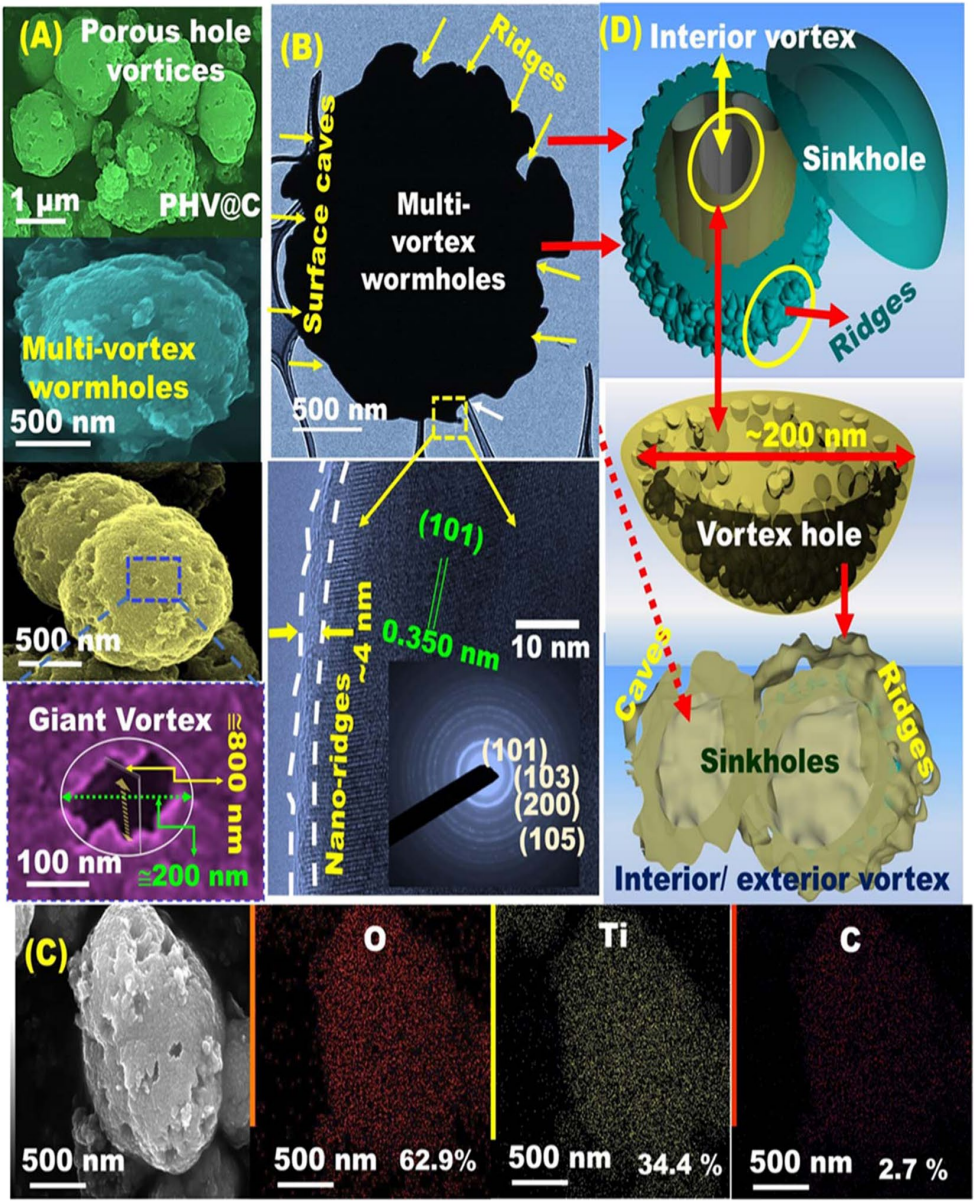
Representative PHV@C anodic meso-geodesics based on low- and high-magnification FESEM images (**A**), HR-TEM micrographs and electron diffraction patterns (**B**), STEM–EDS images and elemental mapping (**C**), and 3D modeling and orientation of spherical TiO2@C anatase structure with massive PHV@C. PHV@C meso-geodesics showed capture caves and ridge surfaces, vortex configurations with connective open meso/macro entrances and cage-like sinkhole core cavities, huge hollowed-out ring bowls, and cliff edges. The unique PHV@C anode module configurations formed full nests distributed and randomly oriented on spherical cuticle and well-defined and homogeneous distribution of active sites along the entire anodic electrode surfaces.

Large-scale meso-geodesics with 3D topographical hierarchy ridges, surface interfaces and vortices of TiO_2_@C (anode), and LiFePO_4_@C (cathode) composites were investigated by a wide range of tools using field-emission scanning electron microscopy (FE-SEM), energy-dispersive X-ray spectroscopy (EDX), and high-resolution transmission electron microscopy (HR-TEM) images (Figs 2 and 3, and Supplementary Information S2 and S3). The engineering designs of large-scale meso-geodesics and diverse ranges of spatial AF@C, CHS@C, and HCS@C as cathodes, and TiO_2_@C (PHV@C) anode hierarchy models were evident. Figures 2 and 3 show the microscopic patterns of geodetically-shaped space sinks with downward/upward surface curvatures, leading to open-ended movement gate options, and diffusible orientations. Along with the primary 3D super-scalable hierarchy, the electrode surface building-blocks with egress/ingress channels, upward/outward curvature cargo-like sphere vehicles, and irregularly located serrated cuticles with abundant V-undulated rigidness, feathery tube pipe conifers, and a band of dagger-shaped needle sticks provide high performance LIB meso-geodesics.

Figures 2(a–d) displays the AF@C meso-geodesic sink cathode with numerous pointed daggers and abundant V-undulated ridge-gate windows along the edge of the 3D surface curvatures. The hierarchical structures of AF@C dagger-shaped needle sticks, convex spheres, and serrated cuticles were leveraged to directly promote (i) the dynamic mobility of electron and Li^+^ ion motion systems, and (ii) the loading upon cargo-like sphere vehicles, as reported with other hierarchical mesoarchitectures^39–43^. In addition, the meso-geodesic hierarchy continuously-harmonized electron/Li^+^ distribution along the geode cavity sink. Figures 1f and 2e reveal the 3D topographic microscopy of the meso-geodesic surface models with dominant HCS@C and CHS@C, respectively. The microscopic images showed a bundle of upward/outward, spheroid-capped gradients in the surface curvature, offering a massive in/out-transport of electron/Li^+^ ions. In particular, the CHS@C showed continuously-twisted anticline/syncline ridge-shaped folds and arch-like shape movements with centrally located curvature space. Such structural vehicle folds are force-driven modulation of the high-energy density of LIB EV (Fig. S2). The CHS@C showed nano-grooves arranged in voids, caves, and mesopore windows and lateral undulations of cladding wave-like movement patterns along the cuticular folding with stratified feathery skins (Fig. S3). Figure 2g shows evidence that the exterior hollow grooves and serrated cuticles of AF@C structure featured abundant V-undulated ridges and conifers, leading to design cathode electrode with multidiffusive gates, high tap density, and wide-range transport pathways of Li^+^ ions. EDX and elemental mapping of large-scale meso-geodesics revealed the good distribution of O: Fe: P: C with distinct weight ratios along all geometrics, (Figs 2d, S3(c,f)). Furthermore, the HR-TEM images obtained on the edge of AF@C, HCS@C, and CHS@C meso-geodesic sink cathodes displayed well-ordered crystal planes with inter-atomic spacing (0.458, 1.01, 0.285, 0.38, and 0.346 nm) corresponding to the (001), (100), (020), (210), and (111) planes, respectively (Figs 1i and 2g, S2g–I, and S3d–f). Selected area electron diffraction (SAED) pattern images indicated that the LFPO@C cathode crystal growth orientation was dominant along the [010] ac plane. The exposed [010] crystal facet is favorable and stable surface energy for fast lithium ion diffusion and high rate capability compared with the other planes^44–47^.

Figure 3 shows the microscopic patterns and 3D objects of anodic [101]-TiO_2_@C (PHV@C) morphology. Given the primary anodic structure tectonics, geodesic PHV@C configurations featured multi-diffusive systems and electron/ion flow gradients. Figure 3A–D shows the formation of PHV@C porous vortex holes with worm pores of 200 nm width and 800 nm depth, enabling a surface creation with caves, ridges, connective open meso/macro entrances, and cage-like sinkhole core cavities^48^. Huge hollowed-out ring bowls, and cliff edges forming full nests were randomly-distributed and oriented PHV@C spherical structures (Fig. 3A-B). The decoration of anode and cathode materials with highly conductive C-shell dots would boost the anisotropic surface heterogeneity and activate surface conductivity and mobility sites. The C-shelled anode/cathode electrodes promote the electron/ion transport and sensitive electron conductivity during the electrons/Li^+^ dynamic diffusion during the discharge (insertion, lithiation)/charge (delithiation, extraction) processes. The HR-TEM lattice pattern images (Figs 2i and 3B) showed the formation of a thin and smoothed 2–4 nm layer of C-shells. A well-dispersed C-dressing along the entire AF@C, HCS@C, and CHS@C surface meso-geodesics are also evidenced from Fourier transform infrared spectroscopy, Raman spectra, thermal analyses, N_2_ isotherms, X-ray photoelectron spectroscopy, and X-ray diffraction characterizations (Figs S4–S9). The [101]-anatase TiO_2_@C crystal structures of the PHV@C anode were evident (see Fig. 3B)^48^.

**Figure 4:**
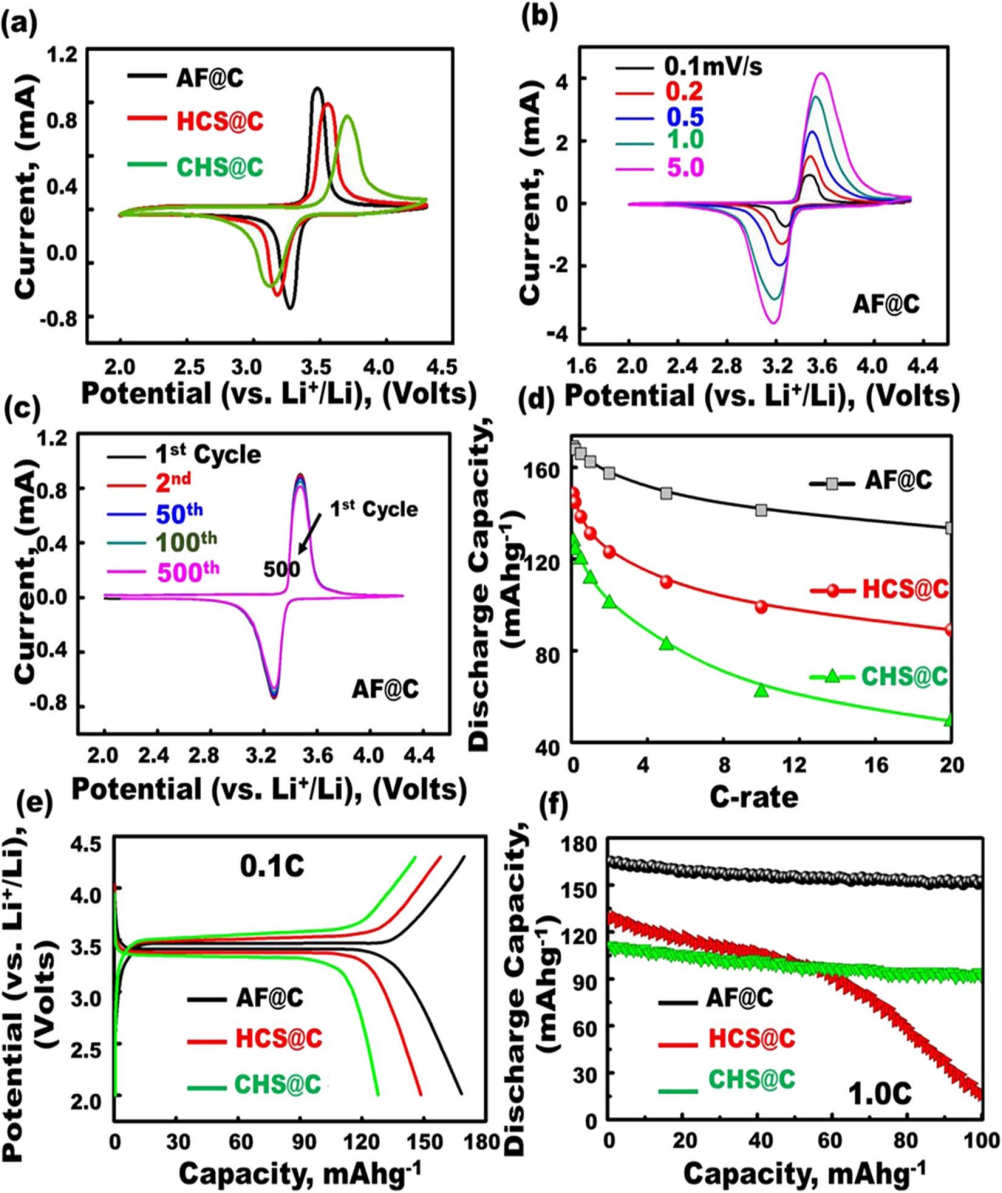
(**a**,**b**,**c**) Cyclic voltammograms obtained from the comprehensive analysis of the electrochemical performance of the anode composites (2032 coin-type half-cell tests with a Li counter electrode). (**a**) CV curves of different structures of geodesic LFPO AF@C with convex spheres and serrated cuticles, HCS@C, and CHS@C as cathode. (**b**) CV curves of AF@C half-cell cathode at different sweep rates (0.1, 0.2, 0.5, 1, and 5 mVs^−1^ and (**c**) CV curves of AF@C half-cell cathode at different cycle numbers 1–500 cycles at 0.1 mVs^−1^. (**d**) First discharge capacity of all LFPO@C of different morphologies (AF@C, HCS@C, and HCS@C) at current rates of 0.1–20 C in half-cell LIBs. (**e**) Charge–discharge voltage profiles of first-cycle AF@C, HCS@C, and HCS@C cathode of different morphologies in half-cell LIBs by using 2032 coin cell with Li foil as the counter electrode at a current rate 0.1 C. (**f**) Cycling performance stability used for AF@C, HCS@C, and HCS@C cathodes at a rate of 1 C for 100 cycles. All electrochemical measurements for half-cell cathodes were operated within a voltage range of 2.0–4.3 V at room temperature.

**Figure 5:**
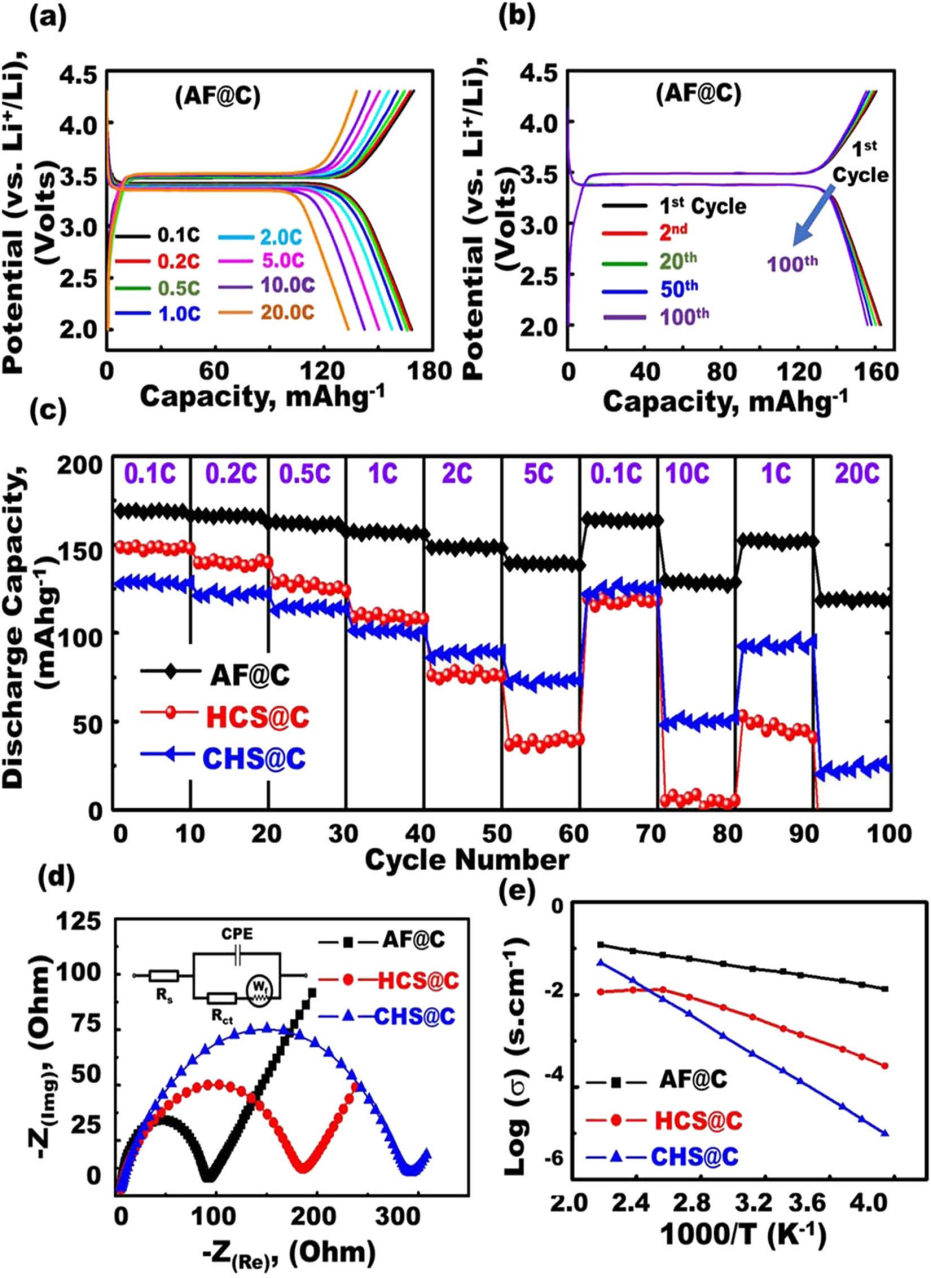
(**a**) First-cycle half-cell AF@C cathode at different current rates from 0.1 C to 20 C. (**b**) AF@C cathode material as a half-cell at a current rate of 1.0 C at different cycles up to 100 cycles. (**c**) Rate capability performance rates of different geodesic LFPO cathodes, such as AF@C with convex spheres and serrated cuticles, HCS@C, and CHS@C, over a range of 2.0–4.3 V and at various current rates from 0.1 C to 20 C. (**d**) EIS results for the prepared AF@C, HCS@C, and CHS@C cathodes of different morphologies. (**e**) Comparison of the temperature dependence of the electrical conductivity of half-cell LIB AF@C, HCS@C, and CHS@C cathodes. All LFPO@C cathodes exhibited good and high conductivity with the AF@C sample along the tested temperature range (~250–455 K). All electrochemical measurements for half-cell cathodes were operated at room temperature 25 °C.

**Figure 6:**
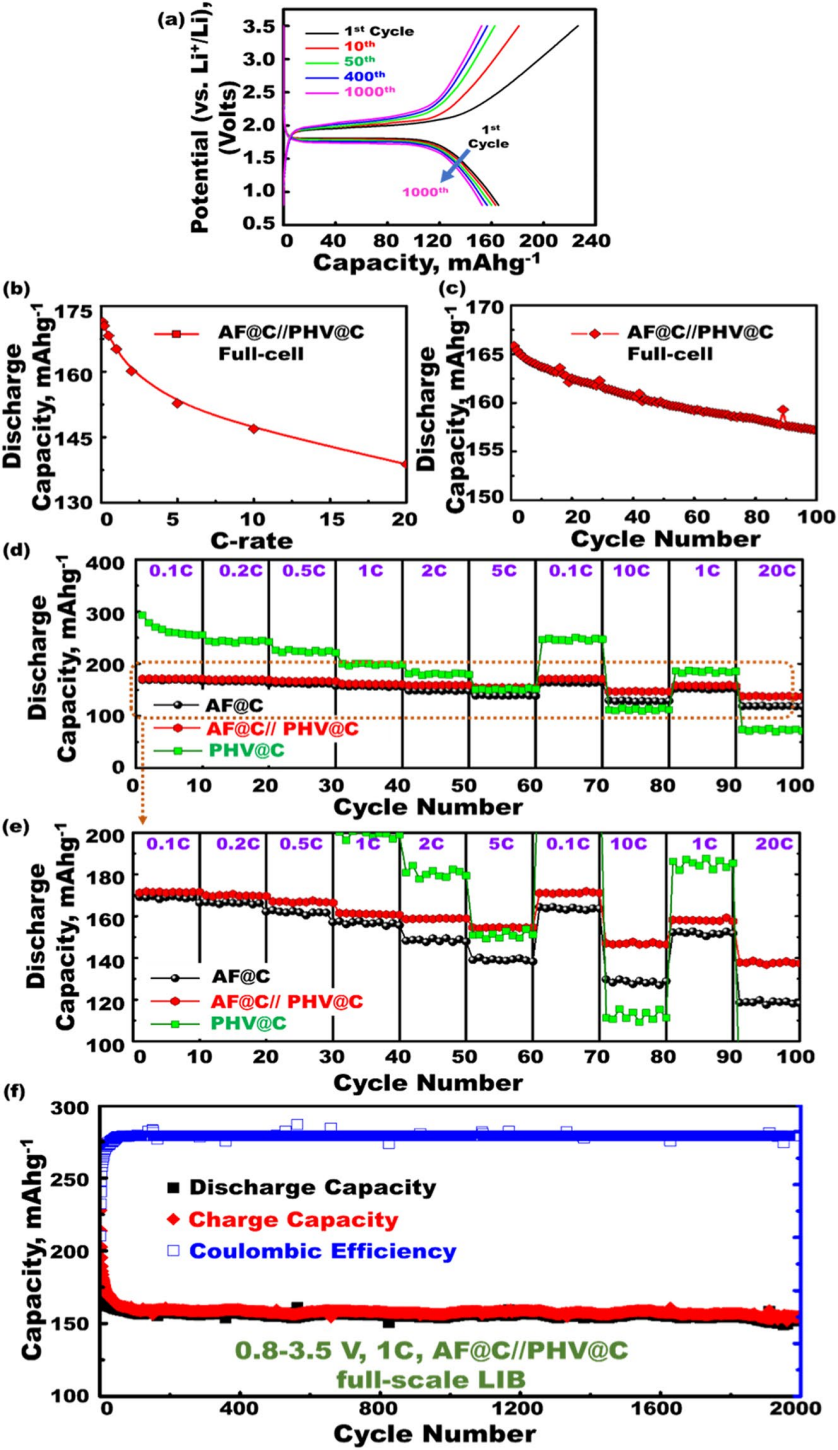
(**a**) Charge–discharge voltage-capacity profiles of AF@C//PHV@C meso-geodesics full-scale LIBs during long-term galvanostatic cycle between 0.8 and 3.5 V at constant current rate of 1 C and room temperature. (**b**) Behavior of specific discharge capacity in mAhg^−1^ versus current C rates at 0.1, 0.2, 0.5, 1, 2, 5, 10, and 20 C and between 0.8 and 3.5 V for the built-in super-hierarchical heterogeneous open geode AF@C (cathode)//anatase TiO2 meso/macro pore spheres PHV@C (anode) full-scale LIB model. (**c**) Cycling performance of AF@C//PHV@C full-scale LIB for the first 100 cycles. (**d**) Performance and rate capability of the AF@C//PHV@C full-scale LIB model over a range of 0.8–3.5 V at various current rates from 0.1 C to 20 C. (**e**) Enlarged selected part of (**d**) for rate capability performance results of spheroid geode built-in AF@C//PHV@C full-scale LIB model. (**f**) Long-term cycling performance (stability) and Coulombic efficiency of full-cell AF@C//PHV@C at a rate of 1 C up to 2000 cycles. All experimental sets were measured at the voltage range 0.8–3.5 V and at room temperature.

Together, the textural parameters featured large surface coverage, atomic-scale dynamic surface arrangement, and meso-geodesics. Furthermore, the encapsulation of the geodesic PHV@C anode//LFPO@C cathode designs in LIB CR2032 coin cells would lead to effective designs. The geodesic modulation of anode and cathode half-scale LIBs, and PHV@C//AF@C full-scale LIB-models with multifunctional surface interfaces, vortices, curvature angularities, spherule cortices, and undulated rigidness may lead to heavily-loaded Li^+^ trucks, non-resisting spread of electrons/Li^+^ loads, and potential occupant diffusions. Due to their rich spatial hollowness complexity and dense-extruded blocks, meso-geodesics-modified PHV@C anode//AF@C, or HCS@C, or CHS@C cathode LIB models are structurally stable to withstand formidable life-use cycles (Figs 4–6 and Supplementary Information S10 and S11).

### Half-cell LIB design based on large-scale AF@C, HCS@C, or CHS@C cathode meso-geodesics

The electrochemical performances of half-cell cathode LIBs based on large-scale AF@C, or HCS@C, or CHS@C meso-geodesics indicate a diverse range of spatial functions and stable transport pathways of electron/Li^+^ movements along the 3D geodetically shaped hollowness orientation, direction axis coordinates, and curvature of space gradients in the LIB cathode models. Together, the structural building blocks of meso-geodesic cathodes are key factors to offer open-ended electron/Li^+^ -ion movement options and a wide range of diffusion orientations as follows (Fig. 1):

(i) multidiffusible dimension pathways along egress/ingress channels,
(ii) multi-accommodation system vacancies through a bundle of cavities, open-mouth sinks, and hollowness in upward/outward curvature cargo-like sphere vehicles,
(iii) multi-accessible directions, which include zigzag, helical, and circular coordinates and irregularly located existing serrated cuticles with abundant V-undulated rigidness, and
(iv) linear gate-transport pathways along the vertical, latitudinal, and longitudinal axis; feathery tube pipe conifers, and a band of dagger-shaped needle sticks.

Figure 4(a) shows the typical first cycle voltammogram profiles of different geodesic LFPO cathodes, such as AF@C with convex spheres and serrated cuticles, HCS@C, and CHS@C, over a voltage window of 2.0–4.3 V versus Li/Li^+^ at scan rate of 0.1 mV/s. The reduction/oxidation peaks were located at 3.27/3.48, 3.18/3.56, and 3.12/3.7 V for AF@C, HCS@C, and HCS@C cathodes, respectively. Figure 4(b) shows the cyclic voltammetry (CV) curves of AF@C for the first cycle at different scan rates of 0.1, 0.5, 1, 5, and 10 mVs^−1^ within the potential range from 2.0 V to 4.3 V. The voltage values for the oxidation and reduction peaks increased and decreased, respectively, with increasing scan rate. Moreover, the absolute current values for the oxidation and reduction peaks increased with increasing scan rate. Figure 4(c) displays the cyclic stability levels of AF@C for the 1st, 10th, 50th, 100th, and 500th cycles at a sweep rate of 0.1 mVs^−1^ within a voltage window of 2.0 V to 4.3 V. The symmetric peak configuration of Fe^2+^/Fe^3+^ within cycles can be easily observed at 3.27/3.48 V for the AF@C cathode, thereby confirming the excellent reversible electrochemical performance of the cathode with Li^+^ insertion (cathodic, reduction, and lithiation)/extraction (anodic, oxidation, and delithiation)^49,50^. The significant coincidence of CV curves during charging/discharging could be attributed to the excellent cycling performance of AF@C, which exhibited high reversible capacity for 500 cycles.

Figure 4(d) presents the typical first cyclic voltammogram profiles for the AF@C, HCS@C, and HCS@C cathodes in LIB half-cells cycled at scan rates of 0.1–20 C between 2.0 and 4.3 V versus Li/Li^+^ at a scan rate of 0.1 mV/s. The first discharge capacity values decreased in the order of AF@C > HCS@C > HCS@C for large-scale meso-geodesic cathodes under the overall scan rates. Figure 4(e,f) show the effect of structural building blocks of different geodesic LFPO cathodes, such as AF@C, HCS@C, and CHS@C, in the charge–discharge cycling performance profiles. Figure 4(e) presents the first charge–discharge cycle of AF@C, HCS@C, and CHS@C half-cell cathodes of different hierarchical structures within the potential region of 2–4.3 V versus Li/Li^+^ at a current rate of 0.1 C. The cells were charged to 4.3 V at 0.1 C and kept at 4.3 V for 1 h. The cells were then discharged to 2.0 V at 0.1 C. The AF@C LIB meso-geodesics would allow the uniqueness of all module configurations per half-cell LIB design direction. The AF@C cathode showed an excellent storage capacity compared with HCS@C and CHS@C cathodes. AF@C exhibited an excellent discharge capacity of 167.2 mAh g^−1^ for the first cycle. This value is higher than that of the HCS@C and CHS@C cathodes, with capacities of 148.8 and 127.6 mAhg^−1^, respectively, at 0.1 C. The excellent discharge capacity of half-cell AF@C cathode may be attributed to the geodetically-shaped electrode surface building blocks that have downward/upward curvature cargo-like sphere vehicles, multi-vast-mouth caves, egress/ingress channels, and convex spheres and serrated cuticles with abundant V-undulated rigidness, feathery tube pipe conifers, and a band of dagger-shaped needle sticks. The building blocks of AF@C electrode surfaces with these multifunctional surface components play a key role in open-ended movement options and retention of diffusion orientations of Li^+^-ion transport through lithiation/delithiation compared with both HCS@C and CHS@C surface cathodes.

Figure 4(f) shows the effect of different geodesic AF@C, HCS@C, and CHS@C cathodes on the superior retention during cycling performance and stability. The AF@C cathode exhibited a retention of 92.4% for its first cycle capacity after 100 cycles at 1 C, whereas 12.5% and 81.9% of their initial capacities were achieved for the HCS@C and CHS@C cathodes after 100 cycles, respectively. The drastic decrease in the capacity of the HCS@C cathode over 50 cycles can be attributed to the presence of huge cracks along the cuticle racks of its spherical particles, (Figs 2(e) and S2). Furthermore, the heating-out of the HCS@C cathode within the charge/discharge cycling caused overloading dissipation and disorder in the electron movement orientations and configurations along the HCS@C particles. Thus, the HCS@C cathode was considered an unstable structure for the LIB models. Compared with other AF@C and CHS@C cathodes, the lower capacity fading in the AF@C-half-cell cathode for the 100th cycle at the rate of 1 C can be attributed to the AF@C electrode robustness and retention of its structurally-mesogeodesic surface features with V-undulated rigidness, serrated cuticles, and open-cave hollow egress/ingress; as such, this AF@C cathode exhibited high electrochemical reversibility during lithiation/delithiation.

As a mode of half-cell cathode LIB meso-geodesics, the geodetically shaped AF@C cathode significantly increased the spatial cycling, rate performance capabilities, resistance, and stability against high temperature (Fig. 5). Figure 5(a) presents the typical first cycle charge/discharge curve of AF@C as a cathode in half-cell LIBs at various scan rates of 0.1, 0.2, 0.5, 1, 2, 5, 10, and 20 C and over a voltage window of 2.0–4.3 V versus Li/Li^+^. Figure 5(a) shows that the AF@C cathode exhibited high discharge capacity over a wide range of current rates from 0.1 C to 20 C compared with half-cell LIB-based HCS@C and CHS@C cathodes. Figure 5(b) illustrates the charge/discharge curves for the AF@C cathode in half-cell LIB at different cycle numbers (1st, 2nd, 20th, 50th, and 100th cycles). This finding emphasizes that the outstanding long-term cycling performance can be ascribed to the stability of the AF@C structure in terms of geodetically shaped downward/upward curvatures, open-ended Li^+^ movement options, and performance diffusion orientations over long cycling of lithiation//delithiation.

In addition to the long-term cycle stability of large-scale meso-geodesic cathodes, the effect of the overall cathode scales of broad-free-access surfaces appeared as nesting object-like “sink,” which eventually affected the cathode half-cell LIB meso-geodesics and their capability performance rate. Cycling performance was tested at different rates (0.1, 0.2, 0.5, 1, 2, and 5 C; 0.1 and 10 C, and then at 1 and 20 C, with 10 cycles at each rate) at room temperature to evaluate the rate capability of the AF@C, HCS@C, and CHS@C cathodes (Fig. 5c). Figure 5(c) shows that the specific capacity decreased with increasing current rate for the AF@C, HCS@C, and CHS@C cathodes in half-cell LIBs. Among the prepared AF@C, HCS@C, and CHS@C cathodes, the 3D super-scalable AF@C meso-geodesic cathode exhibited remarkable performance in terms of outstanding rate capability and long cycle stability with high volumetric energy density at the overall C rate and over 1–100 cycles. The excellent reversible discharge capacity for AF@C cathode half-cell LIB was approximately 118.2 mAhg^−1^ at 20 C after 100 cycles. The reversibly discharge capacity of CHS@C and HCS@C cathodes in half-cell LIBs decreased markedly to 23.7 and 0 mAhg^−1^, respectively, during repeated processed Li intercalation at 20 C after 100 cycles. The drastic drop in the discharge capacity of HCS@C cathode half-cell was attributed to its instability in the overall electron/Li^+^ diffusion scales along/within the broad free-access spheroid surfaces, which appeared when the geodetically shaped HCS@C structure was damaged or cracked with cycles, as evidenced from the cracks along the cuticle walls, (Figs 2(e) and S2).

The evidence of the effective modulation of LIB meso-geodesic half-cell cathodes with 3D topographical hierarchy ridges, surface interfaces, and vortices on the electrochemical performance was further tested by electrochemical impedance spectroscopy (EIS)^51–53^. Figure 5(d) shows the Nyquist plots for AF@C, HCS@C, and CHS@C half-cell cathode materials. The Nyquist graphs show a semicircle in the high-frequency region and a slanted line-in the low-frequency region. The EIS results can be illustrated based on the equivalent circuit shown in the inset of Fig. 5(d). The equivalent circuit consisted of (i) electrolyte resistance (R_s_ is the intercept impedance on the real axis, which corresponds to the solution resistance), (ii) charge transfer resistance (R_ct_ is due to the electrochemical interaction at the electrode/electrolyte interface and particle/particle contact), (iii) constant phase element, and (iv) Warburg impedance of Li^+^ diffusion into the electrode material (W_f_ is related to the low-frequency region of the straight line)^44,51,53^. The semicircular arc in the highest frequency range is relative to the R_ct_ value. The R_ct_ is approximately equal to the diameter of the semicircle on the Z real axis. The smallest semicircle diameter and the minimum R_ct_ value were observed for AF@C-cathode half-cell compared with other LIB meso-geodesic half-cell cathodes. The minimum R_ct_ value of Af@C cathode indicates the excellent electron/ion transfer kinetics along its cuticles during lithiation/delithiation.

Using extensive experiments, we assumed that the well-ordered decoration and sustainable coating of C-shell dressers along geodetically shaped AF@C, HCS@C, and HCS@C cathodes or PHV@C anode assisted the retention of fast kinetics of electron/Li^+^ transport during lithiation/delithiation. In general, the C-shell dresser anode/cathode would enhance the electronic conductivity and surface transport functionality. The surface functions and hierarchal mesogeodesics of LFPO@C cathodes and PHV@C anode enabled excellent spatial cycling, rate performance capabilities, resistance, and stability against high temperature compared with pristine AF-, HCS-, and CHS cathodes or PHV anode modulated in half-cell or full-scale LIB modules. Therefore, in such remarkable 3D super-scalable meso-geodesic LIB model, the encapsulated C-shell dressers (approximately 3–5 nm) play an important role in the following;

(i) the robustness of structurally-geodesic cathode/anode morphologies with multifunctional surface interfaces, vortices, curvature angularities, spherule cortices, cave blocks, and undulated rigidness;
(ii) improvement of the sustainability of atomic-scale arrangement against cycles;
(iii) stability of meso-geodesic orientations, configurations, functional building block egress/ingress, and rich spatial distribution complexity during lithiation/delithiation; and
(iv) protection of the meso-geodesic electrode cuticles from degradation against the heat formed within multiple charge/discharge cycles.

The temperature dependence functionality and electrical conductivity of geodetically shaped AF@C, HCS@C, and HCS@C half-cell cathodes were studied to explore the retention of the primary structural cathode tectonics against thermal treatment, Fig. 5(e). All the tested geode half-cell cathodes exhibited good conductivity at room temperature (298 K). The AF@C cathode in half-cell LIB module is a promising candidate for LIB fabricator; it attains its functionality at a wide range of temperature (~250–455 K).

### Geodetically-shaped vortices of PHV@C half-cell anode LIBs

This study explored the effectiveness of PHV@C anode geodesics designated with multifunctional surface interfaces, vortices, curvature angularities, spherule cortices, cave blocks, and undulated rigidness, which provide massive channel gates, broad free-access pathways, and facile Li^+^ diffusion and transport, in the integral and outstanding half-scale LIB-model. First, we studied the electrochemical performance of half-cell PHV@C anode in the LIB design (Supplementary Information S1). Figure S10(a) shows the key clues of half-cell PHV@C anode in the charge–discharge potential curves of the first cycle at different rates of 0.2, 1, 5, 10, and 20 C. The galvanostatic charge/discharge profiles of the anatase PHV@C anode in half-cell LIB configurations in the voltage region from 1 V to 3 V versus Li/Li^+^ were investigated. Each discharge curve was divided into three bands. The first region of discharge was due to the formation of solid solution (insignificant quantity of Li^+^ were inserted into the PHV@C electrode) and specified with rapid fall in the potential from open-circuit voltage (~3 V) to ca. 1.7 V versus Li. The second discharge zone exhibited a long distinct flat plateau at ca. 1.7 V because of lithiation, which can bind half of the vacant octahedral sites of anatase structure of TiO_2_-PHV. The third discharge stage displayed a long gradual decrease in the potential after the plateau zone from 1.7 V toward a cut-off voltage of 1.0 V versus Li. This result can be attributed to the substantial lithiation in the interior structure of PHV@C spheroids, meso/macro holes, and vortex pores along its surfaces (i.e., interfacial pocket storage). The charge profiles also exhibited three regions. The first region showed the increased capacity from 1 V to 1.96 V due to monotonic delithiation, which was directly followed by the second region of continued Li^+^ extraction through the plateau regime. Finally, the third region indicated a curved solid solution regime toward 3.00 V. Figure S10(b) illustrates the first-cycle discharge specific capacity values of the half-cell PHV@C anode at current rates within 0.2 C to 20 C. Figure S10(c) displays the Nyquist plot of the EIS results for the half-cell PHV@C anode. The smallest semicircle diameter of the half-cell PHV@C anode indicates that the anode surfaces featured multidiffusive systems for electron/ion flow gradients. It is clearly obvious that the stability of structural building blocks of PHV@C anode with multiple characteristics such as porous vortex holes (200 nm width and 800 nm depth) and worm-pore- and nesting object-like “sink,” leads to excellent electron/ion transfer kinetics during lithiation/delithiation (see Fig. 3)^29–33^.

To evaluate the influence of geodetically shaped ridge surfaces and vortex configurations of PHV@C structures on the rate capability performance over a range of 1.0–3.0 V for the PHV@C anode, we studied the discharge cycling performance at different rates of 0.1, 0.2, 0.5, 1, 2, and 5 C, 0.1 and 10 C, and 1 and 20 C with 10 cycles at each rate until 100 cycles at room temperature (Fig. S11). The rate capability finding proves that the specific capacity of half-cell PHV@C anode decreased with increasing current rate. The capacity of half-cell PHV@C anode decreased to 89.2 mAhg^−1^ at the rate of 20 C and after 100 cycles during repeated Li intercalation. However, the high specific capacity retention of the PHV@C anode compared with the HCS@C and CHS@C cathodes in half-cell LIBs indicates the effectiveness of the homogeneous distribution of active sites along the surface sinkhole core cavities, vortices, huge hollowed-out ring bowls, and cliff edges of PHV@C anodic electrodes on attaining electron/ion flow gradients during charge/discharge cycles^30–32^.

### 3D super-scalable model of full-scale anode//cathode LIB meso-geodesics

As primary structural tectonics, geodesic AF@C cathode, and PHV@C anode are probably the visible options to study their capability to function in simultaneous, full-scale LIB models (Figs 1 and 6). On the basis of the key advances of the designated half-cell-based PHV@C (anode) and AF@C (cathode) in LIB designs, we integrated the anode//cathode full-scale hybrid design in a model called 3D-superscalable built-in AF@C//PHV@C LIB meso-geodesics (Figs 4–6 and S10, S11). A powerful built-in 3D super-scalable model of full-scale LIB-based AF@C//PHV@C meso-geodesic building blocks was designated for the first time in 2032 coin-type full-scale tests. The heterogeneous open-geode AF@C (cathode) and anatase meso/macropore spherical PHV@C (anode) materials were loaded on Al and Cu foil as counter electrodes, respectively, under supreme conditions (Figs S1 and Table S1). Our full-scale LiFePO_4_@C//TiO_2_@C (cathode//anode) pouch LIB model was designed with six stacked cathode layers. The layer sequences were formulated with 10 sides loaded on the Al-foil (10 µm) positive (P) collector. In turn, the number of stacked layers of anode was five with 10 sides loaded on the Cu-foil (8 µm) negative (N) collector. A suitable combination of N-electrode (PHV@C anode) and P-electrode (AF@C cathode) materials may lead to cost-effective and high-capacity LIBs. In our built-in AF@C//PHV@C LIB meso-geodesics, reasonable optimization of the active mass of N and P electrodes (i.e., capacity balancing of N/P ratio) is required to realize the equal discharge specific capacities of both N and P electrodes (i.e., (N:P)_Cap_ ratio = 1:1) for the safe, long-term, practical, and high performance of full-cell LIBs during their operation (Table S2). In this regard, our proposed 3D super-scalable full-scale LiFePO_4_//TiO_2_ cathode//anode stacked-layer pouch LIB model was fabricated under optimized mass loading and ((N:P)_Cap_ ≈ 1.02–1.1 :1.

Figure 6(a) presents the charge–discharge voltage-capacity profiles of AF@C//PHV@C meso-geodesic full-scale LIBs under long-term galvanostatic cycling up to 1000 cycles, at voltage between 0.8 and 3.5 V, at a constant current rate (1 C), and at room temperature. The first-cycle charge and discharge capacities were 273.9 and 165.8 mAh g^−1^, respectively, referring to 60.5% of the initial Coulombic efficiency. The Coulombic efficiency improved to 91% after 10 cycles and then reached approximately 100% after 50 cycles. The irreversible capacity loss in the first 10 cycles was possibly due to the electrolyte decomposition and formation of the stable solid−electrolyte interface (SEI) layer on the PHV@C anode. Figure 6(a) also shows that the average working voltage of the LIB is ~1.8 V, providing a specific energy density of 297 Wh kg^−1^ for the LFPO (AF@C) cathode. Based on the experimental findings, the mass fraction analysis of a commercial pouch LIB cell was used for estimating the mass fraction of individual cell components (Table S1). Specifically, the mass fraction of AF@C cathode material in a LIB cell is approximately 40%, which leads to a specific energy density of 118.8 Wh kg^−1^ for the AF@C//PHV@C full-scale pouch LIB meso-geodesics (Supplementary Information S12). This specific energy density (i.e., 118.8 Wh kg^−1^) for the AF@C//PHV@C full-cell was supported the salutary limit imposed by the driving range requirement for EVs^1,2^. In our study, the high volumetric energy density featured the large-scale EVs using AF@C//PHV@C full-scale system agreed with the cylindrical shaped 18650 model as a preferable design compared with the pouch cell (Supplementary Information S13). On the basis of pack parameters and module configuration studies (Supplementary Information S13D), 9800 cells were proposed (18650-AF@C//PHV@C) for full-scale cells to establish practical full-packed LIB EVs (i.e., pack stored energy of 50 kWh and module voltage of 180 V) (see Table S3). Furthermore, the mass ratio between the total number of cells and the whole pack (i.e., C/P) of the proposed (18650-AF@C//PHV@C) full-scale cells was 68%, which is notably reasonable value compared with that of Tesla Co. Ltd (C/P = 63%) (Table S3).

The specific energy density values for the (LFPO-based cathode//TiO_2_-based anode) AF@C//PHV@C meso-geodesic full-scale LIBs were 118.8 and 135 Wh kg^−1^, according to the practical and theoretical investigation, as shown in Supplementary Information S12. The key clue for the low practical capacity compared with the theoretical value is that all the Li^+^ cannot be removed from the lattice site of the actively host material-based electrodes. The remaining Li^+^ can also be removed on the value above the cutoff potential, leading to non-accessible sites. The finding indicates the effect of geodesics with multifunctional surface interfaces, vortices, curvature angularities, spherule cortices, cave blocks, and undulated rigidness on the retention of multidiffusive systems, electron/ion flow gradients, and channel gates during the cycles of full-scale anode//cathode LIB meso-geodesics.

The behavior of specific discharge capacity in mAhg^−1^ versus current C rates was studied from 0.1 C to 20 C for the designated full-cell AF@C//PHV@C LIB meso-geodesics to determine the most effective anode/electrode designs for building substantial LIB objects (Fig. 6(b)). An excellent discharge capacity was observed over all the range of current rates at 0.1, 0.2, 0.5, 1, 2, 5, 10, and 20 C and between the voltages 0.8 and 3.5 V (Fig. 6(b)). The discharged capacities decreased with increasing C rate and were maintained at 137.8 mAhg^−1^ at 20 C. Figure 6(c) shows evidence of retention of cycling performance and stability of full-cell AF@C//PHV@C LIB meso-geodesics. The built-in AF@C//PHV@C full-scale LIB-model exhibited a retention of 91.5% in the first-cycle capacity after 100 cycles at 1 C. A set of experimental evaluations of the capability performance rates of full-cell AF@C//PHV@C (cathode//anode) LIB meso-geodesics at a range of 0.8–3.5 V was studied at different C rates (0.1, 0.2, 0.5, 1, 2, and 5 C, 0.1 and 10 C, and 1 and 20 C) with 10 cycles at each rate up to 100 cycles, and at room temperature (Fig. 6d,e). In general, the specific capacity of full-cell AF@C//PHV@C LIB meso-geodesics decreased with increasing current rate. This finding indicates that full-cell AF@C//PHV@C LIB meso-geodesic cuticles with multifunctional surface interfaces may open non-prescriptive cycle usages, non-resisting spread of electrons/Li^+^ loads, potential occupant diffusions (see Supplementary Information S14). The AF@C//PHV@C LIB meso-geodesics substantially maintained excellent electronic conductivity and reduction of Li^+^ diffusion paths/distances on the cathode//anode surfaces within the cycles^1,2,54^.

To explore the nonprescribed geometric LIB models that are structurally stable to withstand formidable life-use cycles and can be readily adjusted to high levels of heavily Li^+^ truck loads, we aim to investigate cycling performance, long-term stability, and Coulombic efficiency of AF@C//PHV@C LIB meso-geodesics. The behavior of specific discharge capacity in mAhg^−1^ versus current C rates was studied at 1 C for up to 2000 cycles in the potential region from 0.8 V to 3.5 V versus Li/Li^+^ at room temperature (Fig. 6f). The large-scale full-cell AF@C//PHV@C LIB meso-geodesics offered (i) 91.5% retention of the first discharge capacity of 165.8 mAhg^−1^ after 2000 cycles, (ii) Coulombic efficiency of ~99.6% at the rate of 1 C and at room temperature, and (iii) high specific energy density of ≈119 Wh kg^−1^. Our LIB meso-geodesic module configurations may align perfectly with the requirements of the energy density limit mandatory for long-term EV driving range and the scale-up commercial manufactures.

**Figure 7:**
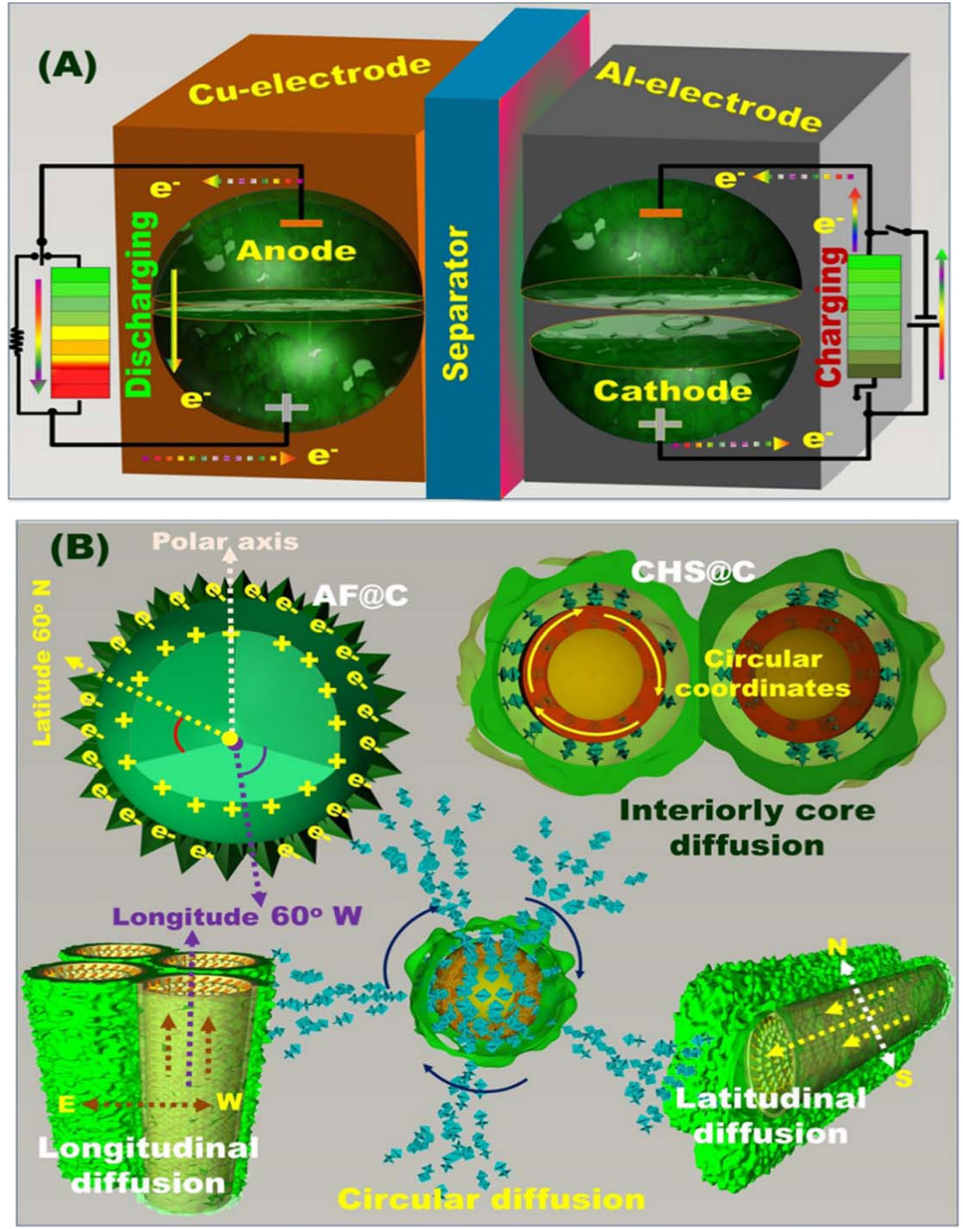
(**A**) Systematic design of long-term lithiation/delithiation (discharge/charge) cycling. (**B**) Interior/exterior hollow electron/ion diffusion pathways along the 3D mesopores, hollow cavities, open-mouth sinks, axial directions, and axis coordinates of hierarchy AF@C decorated with meso-geodesic cavity and needle dendrites along the 3D anisotropic hollow surfaces and HCS@C cathodes. (**A**,**B**) Full-scale LIB cell configuration of TiO_2_@C (PHV@C, anode)//LFPO@C (cathode) electrodes markedly guided for excellent long-term cycle stability for 2000 cycles at rate of (1C). In full-cell LIB design fabricated with full 3D hollow orientation of anode/cathode, the structural hierarchy model with a bundle of upward/outward spheroid-capped gradients designed in different surface curvatures and multi-accessible directions, including zigzag, helical, circular, latitudinal, and longitudinal axis, offers the key clues for the capacity retention of LIBs. The continuous function and stability of transport pathways of electron/Li^+^ ions movements after 2000 cycles and simultaneous trapping/detrapping of Li^+^ ions during the lithiation/delithiation (discharging/charging) cycling for full-cell-based (PHV@C) (anode)//(LFPO@C) (cathode) were evident.

Wide range cycles of full-cell AF@C//PHV@C LIB meso-geodesics demonstrated ~100% Coulombic efficiency. As a merit of the LIB model, for up to 2000 cycles, the proposed full-scale AF@C//PHV@C LIB meso-geodesics significantly exceeds the spatial rate performance capabilities, long-term cycling performance, excellent capacity retention, specific energy density, and average Coulombic efficiency like that of the most widely used common-scale LIB fabricators, which would complicatedly increase the LIB costs. The overall geodetically shaped scales retained broad free-access surfaces appearing as nesting object-like sink. The meso-geodesic surface functionality leads to outstanding stability of excellent electrochemical performances after 2000 cycles, ~100% Coulombic efficiency, high capacity at high rate capability, and long cycle life (Table S4). The occupied anode//cathode meso-geodesics with 3D topographic orientations, configurations, and functional open-ended mouth caves, vortices, curvature angularities, spherule cortices, and undulated rigidness may lead to stable charge/discharge participatory modules for future energy storage systems^55,56^. Together, the AF@C//PHV@C LIB meso-geodesics provide fully-cycled dynamics, affordable on/off-site storage pocket modules and lodges, and super-large gate-in transport of electron/Li^+^ ions. The LIB meso-geodesics are projected to be the force-driven modulation of high energy density of LIB EVs (Fig. 7)^4^.

## Conclusion

The integration of full-scale LIB anode/cathode with 3D topographical hierarchy ridges, surface interfaces, and vortices was performed to achieve a superior energy density with outstanding durability and Coulombic efficiency, high capacity, and excellent capacity retention. The unique features of AF@C cathodic and PHV@C anodic materials provide visible options to study their functions in simultaneous and full-scale LIB CR2032 coin cells. The 3D super-scalable AF@C//PHV@C cathode//anode LIB meso-geodesic full-scale LIB model showed substantial evidence of long-term stability and excellent capacity retention of 91.5% at Coulombic efficiency reaching 99.6% for 2000 cycles at 1 C rate. The built-in AF@C//PHV@C full-scale LIB model meso-geodesics successfully provided a high specific energy density of ≈119 Wh kg^−1^. The structural building blocks of meso-geodesics provide broad free-access surfaces appearing as nesting object-like sink, fully-cycled dynamics, affordable on/off-site storage pocket modules and lodges, hovering electron density for high-speed discharge rates, and super-large gate-in transport of electron/Li^+^ ions. To the best of our knowledge, the built-in LIB meso-geodesic hierarchy is a promising model for energy storage applications.

## Supplementary information

Meso/macroscopically multifunctional surface interfaces, ridges, and vortex-modified anode/cathode cuticles as force-driven modulation of high-energy density of LIB electric vehicles
