# Supplementary material for: Meso/macroscopically multifunctional surface interfaces, ridges, and vortex-modified anode/cathode cuticles as force-driven modulation of high-energy density of LIB electric vehicles
Source: Sci Rep. 2019 Oct 11;9:14701. doi: 10.1038/s41598-019-51345-z (PMC6789099; doi:10.1038/s41598-019-51345-z)
Supplement: Supplementary file 1 — Meso/macroscopically multifunctional surface interfaces, ridges, and vortex-modified anode/cathode cuticles as force-driven modulation of high-energy density of LIB electric vehicles [file 41598_2019_51345_MOESM1_ESM.pdf]

Meso/macroscopically multifunctional surface interfaces, ridges, and vortex-modified anode/cathode cuticles as force-driven modulation of high-energy density of LIB electric vehicles

H. Khalifa[1], S.A. El-Safty[1] (✉), A. Reda[1], M.A. Shenashen[1], M.M. Selim[2], O.Y. Alothman[3], N. Ohashi[4]

[1] *National Institute for Materials Science (NIMS), Sengen 1-2-1, Tsukuba, Ibaraki 305-0047, Japan.*

[2] *Department of Mathematics, Al-Aflaj College of Science and Human Studies, Prince Sattam Bin Abdulaziz University, Al-Aflaj 710-11912, Saudi Arabia*

[3] *Chemical Engineering Department, College of Engineering, King Saud University, P.O. Box 800, Riyadh, 11421, Saudi Arabia*

[4] *Research Center for Functional Materials, National Institute for Materials Science, 1-1 Namiki, Tsukuba, Ibaraki, 305-0044, Japan.*

***** Correspondence and requests for materials should be addressed to S.A.El-Safty (Email: sherif.elsafty@nims.go.jp)

S1. Materials, Characterizations and Electrode Synthesis

A- Chemicals used for fabrication of large-scale anode/cathode meso-geodesics

All chemicals were of analytical grade and were used without further purification. Lithium fluoride (LiF), iron (III) nitrate nonahydrate ($Fe(NO_3)_3{\cdot}9H_2O$), titanium (IV) ethoxide ($Ti(OC_2H_5)_4$), hydrochloric acid (HCl, 2mol/L), hydrogen peroxide solution (H_2O_2) and ethanol(C_2H_5OH) were purchased from Sigma–Aldrich Company, Ltd., USA. Phosphoric acid (H_3PO_4) was supplied from Tokyo Chemical Industry (TCI) Company, Ltd., Tokyo, Japan. Ethylene glycol ($C_2H_6O_2$), and lithium carbonate (Li_2CO_3) and lithium chloride ($LiCl_3$) were purchased from Nacali Tesque Company, Ltd., Kyoto, Japan. All the chemicals added in stoichiometric ratios, TiO_2 and LFPO

compositions were synthesized by the hydrothermal method. Carbon coating process was carried out by microwave irradiation technique.

B- Characterization large-scale anode/cathode meso-geodesics

X-ray diffraction (XRD) characterization was performed to investigate the crystallographic information of geodesic LFPO artichoke flower hollows (AF@C) that have convex spheres and serrated cuticles, conjugated hollow spheres (CHS@C), and hairy coconut sphere (HCS@C) as cathode, and anodic TiO_2@C porous nano-sink holes-like vortices (PHV@C-anode) hierarchy-annealed samples using 18 kW diffractometer (Bruker D8 Advance X-ray diffractometer) at scan rate of 10°/min with CuKα-X-radiation (λ = 1.54178 Å) at 30 kV and 10 mA.

Scanning electron microscopy (SEM) and transmission electron microscopy (TEM) were used to investigate the details of the 3D topographic structures of geodesic LFPO@C of different morphologies (AF@C, HCS@C, and HCS@C) cathode, and anodic TiO_2@C (PHV@C-anode) hierarchy. The anode/cathode structures were investigated by field emission-type scanning electron microscope FE-SEM (Jeol JSM-Model 7000F, JEOL Ltd) at 20 kV. This SEM is provided by Schottky (thermal type) field emission electron gun. Analysis material was fixed onto the FE-SEM stage using carbon tape before insertion into the FE-SEM chamber. The ion sputter (Hitachi E-1030) was used to deposit thin-layered Pt films on electrodes at 25 °C. High-resolution transmission electron microscopy (HRTEM) images, electron diffraction (ED), scanning transmission electron microscopy (STEM) were performed at atomic-level imaging, structural and chemical analysis field emission-type TEM (JEM-ARM200F). The accelerating voltage can be adjusted to 80, 120 or 200 kV, depending on the specimen material and the purpose of observation. Energy-dispersive X-ray spectroscopy (STEM-EDS) was carried out during TEM measurements with high-resolution elemental mapping to investigate the chemical contents of the geodesic LFPO@C of different

morphologies (AF@C, HCS@C, and HCS@C) cathode, and anodic TiO_2@C (PHV@C-anode) hierarchy by using a 200 kV TEM (JEOL 2100F, JEOL Ltd) field emission-type transmission electron gun microscope. The samples were dispersed in ethanol solution using an ultrasonic radiation, and then dropped on a copper mesh and vacuum dried for 20 min prior to inserting the samples into the HR-TEM and STEM-EDS columns.

The surface properties of geodesic LFPO@C of different morphologies (AF@C, HCS@C, and HCS@C) cathode, and anodic TiO_2@C (PHV@C-anode) hierarchy including the pore structure distribution and surface area were estimated by N_2 adsorption–desorption isotherms at 77 K using a BELSORP36 analyzer (JP. BEL Co., Ltd.). The samples were thermally treated at 200 °C for at least 6 h under N_2 atmosphere. The specific surface area (S_{BET}) was calculated using the Brunauer–Emmett–Teller (BET) method with multipoint adsorption data from the linear section of the N_2 adsorption isotherm. The pore size distribution was determined using nonlocal DFT (NLDFT).

The thermal stability of geodesic LFPO@C artichoke flower hollows (AF@C) that have convex spheres and serrated cuticles, conjugated hollow spheres (CHS@C), and hairy coconut sphere (HCS@C) as cathode, and anodic TiO_2@C porous nano-sink holes-like vortices (PHV@C-anode) hierarchy is crucial for establish outstanding cycling performance, stability and Coulombic efficiency for powerful 3D super-scalable model of full-scale anode//cathode LIB meso-geodesics under wide range of temperatures and after numerous cycles (ageless life time). To investigate the thermal stability of large-scale cathode//anode meso-geodesics, the weight content loss of heterogeneous spheroid cathode composites were determined from the weight loss curve measured under simulated air atmosphere on thermo-gravimetric and differential scanning calorimetry (TG/DSC) instrument TG/DSC-60 (Shimadzu, Japan) with a heating rate of 10 ^oC min^{-1}.

The formulation of 3D super-scalable model of full-scale anode//cathode LIB meso-geodesics was investigated by using X-ray photoelectron spectroscopy (XPS), Raman spectroscopy, and

Fourier transform infrared spectroscopy, respectively. For instance, X-ray photoelectron spectroscopy (XPS) analysis (0-1400eV) of geodesic LFPO@C of different morphologies (AF@C, HCS@C, and HCS@C) cathode, and anodic TiO_2@C (PHV@C-anode) hierarchy was conducted on a PHI Quantera SXM (ULVAC-PHI) instrument (Perkin–Elmer Co., USA) equipped with Al K_α radiation as an X-ray source for excitation (1.5 mm × 0.1 mm, 15 kV, 50 W) under a pressure of 4×10^{-8} Pa.

Raman spectroscopy (HR Micro Raman spectrometer, Horiba, Jobin Yvon) of geodesic LFPO@C of different morphologies (AF@C, HCS@C, and HCS@C) cathode, and anodic TiO_2@C (PHV@C-anode) hierarchy was conducted using an Ar ion laser at 633 nm. A charge coupled device (CCD) camera detection system and the LabSpec-3.01C software package were used for data acquisition and analysis, respectively. To ensure the accuracy and precision of the Raman spectra, 10 scans of 5s from (500-2000 cm^{-1}) were recorded. The chemical compositions of the 3D super-scalable model of full-scale anode//cathode LIB meso-geodesics were analyzed by Fourier transform infrared spectroscopy (ATR-FTIR, Spectrum 100, Perkin-Elmer, Inc., USA).

C- Control design of built-in 3D super-scalable model of full-scale anode//cathode LIB meso-geodesics

All consumed chemicals for new generation of 3D super-scalable model of anode//cathode LIB meso-geodesics in full-scale LIB-model, half-scale AF@C, HCS@C, and CHS@C meso-geodesic cathodes, and PHV@C anode LIB- models have high analytical grade. All chemicals have been used without further purification. For instance, lithium hexafluoro-phosphate $LiPF_6$, carbon black and polyvinylidene fluoride (PVDF) are from Sigma–Aldrich Company, Ltd., USA. N-methyl-2-pyrrolidone (NMP) from Tokyo Chemical Industry (TCI) Company, Ltd., Tokyo, Japan. To perform the electrochemical measurement, Li-ion intercalation was performed using CR2032 coin-cells that

assembled in a glovebox under pure Ar-gas. Within a design of half-cell positive LFPO@C-cathode/negative PHV@C-anode electrodes, we used lithium foil used as reference and counter electrode to control the electrochemical performances of the half-scale geodesic LFPO@C artichoke flower hollows (AF@C), conjugated hollow spheres (CHS@C), and hairy coconut sphere (HCS@C) as cathode, and anodic TiO_2@C porous nano-sink holes-like vortices (PHV@C-anode) hierarchy. In turn, we designated AF@C//PHV@C as anode//cathode full-cell LIBs for electrochemical measurements of full- system level design of full-scale LIB-EV model system (Figure S1).

To investigate the electrochemical performance of the fabricated 3D super-scalable model of full-scale AF@C//PHV@C cathode//anode LIB meso-geodesics full-scale LIB-model, half-scale geodesic LFPO@C artichoke flower hollows (AF@C) that have convex spheres and serrated cuticles, conjugated hollow spheres (CHS@C), and hairy coconut sphere (HCS@C) as cathode, and anodic TiO_2@C porous nano-sink holes-like vortices (PHV@C-anode) hierarchy, galvanostatic charge/discharge characteristic were measured using multichannel battery system (LAND CT2001A, Wuhan, China). Cyclic voltammetry (CV) measurements of the cells were tested using (CHI 660c electrochemical workstation). Electrochemical impedance spectroscopy (EIS) was performed using (Zennium/ZAHNER-Elektrik GmbH & CoKG, controlled by Thales Z-3.0 software –frequency range from 0.1 Hz to 1 MHz). All the electrochemical measurements were done at room temperature. Based on the mass fraction analysis of a commercial pouch LIB cell, the mass fraction of the active cathode material in a LIB cell was approximately 40% (Table S1 for the estimated mass fraction of individual cell components)[1] . The mass fraction can be used to determine the specific energy density for the AF@C//PHV@C meso-geodesic full-scale LIBs.

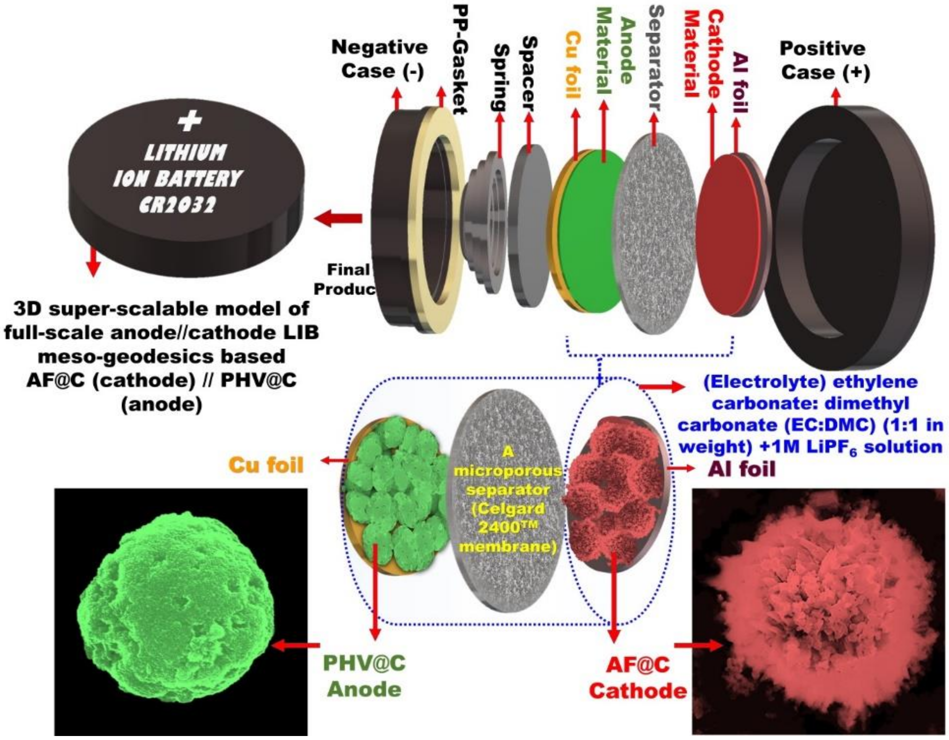

Fig. S1. Illustration of LIB CR2032-type coin cell assembly. Schematic design of formulation of heterogeneous built-in PHV@C (anode) and AF@C (cathode) designated in coin-cell CR2032-type full-scale LIB-model.

Table S1 *Mass fraction of individual components used in the pouch full-cell LIB.*

Pouch cell components	Mass (%)	Mass (g)	Active Material
Cathode	39.5	2.37	75% $LiFePO_4$ cathode active material (1.778g); 15% carbon black (0.36g); 10% PVDF as a binder (0.24g)
Anode	20.5	1.23	75% TiO_2 anode active material (0.923g); 15% carbon black (0.18g); 10% PVDF as a binder (0.123)
Al foil collector	3.9	0.23	
Cu foil	8.2	0.5	
Separator	3.1	0.19	
Electrolyte	15.7	0.94	
Packaging	9.1	0.54	
**total**	100	6	

D. Full-scale $LiFePO_4$//TiO_2 cathode//anode stacked-layer pouch LIB-model

Full-scale $LiFePO_4$//TiO_2 cathode//anode stacked-layer pouch LIB model was prepared with dimensions of 35 (width), 55 (length), and ~2–2.5 mm (thickness). Table S1 illustrate the weight fraction calculation for the pouch cell components. Table S1 provides the active mass of the cathode (1.78 g, i.e., 1778 mg) and anode (0.923 g, i.e., 923 mg). Our full-scale $LiFePO_4$//TiO_2 cathode//anode pouch LIB model was designed with a number of six stacked cathode layers with 10 sides loaded on the Al foil (10 µm) positive (P) collector. In turn, the number of stacked layers in the anode is five with 10 sides loaded on the Cu foil (8 µm) negative (N) collector.

To optimize the full-cell $LiFePO_4$//TiO_2 cathode//anode stacked-layer pouch LIB-model, the electrode area was considered as (3*5=15 cm^2) and (3*4.75 = 14.3 cm^2) for the cathode and anode, respectively. Therefore, the total areas of the cathode and anode in the cell were 150 and 143 cm^2, respectively. In addition, the mass loadings of active cathode and anode materials were 11.85 and 6.49 mg/cm^2, respectively.

S2-S3. Morphological control of geodetically-shaped cathode hierarchy

In the fabrication of large-scale meso-geodesics, we have pursued a striped procedure based on time-dependent chemical-control-hydrothermal approach allows us to fabricate geodetically-shaped LFPO cathodes with various morphologies. Specific anticipating arrangement factors and structured building blocks control the structural tectonics with 3D- multidirectional gates of geodesic LFPO artichoke flower hollows (AF@C) that have convex spheres and serrated cuticles, conjugated hollow spheres (CHS@C), and hairy coconut sphere (HCS@C) as cathode, and anodic TiO_2@C porous nano-sink holes-like vortices (PHV@C-anode) hierarchy. These hierarchal built-in LIB meso-geodesic designs based on a series of irregular surface ripples, bumps, and undulations and

anticlines affected diffusion capability, and electrochemical reactivity in potential half and full-scale LIBs.

To fabricate 3D-topographical hierarchy cathode composites as positive electrode materials, we tailored the synthesis protocol by varying the Li-precursor (chloride, fluoride and carbonate, respectively), while keep the molar ratio 3:1:1 (Li:Fe:P) constant, under special control of time-dependent seed growth and high-temperature of composites. To understand the building-growth of LFPO crystals with various 3D-morphological structures, typical variables such as nucleation-directing agent, and reagents insertion rate such as aqueous ethylene glycol and ethanol may lead to control the nucleation of surface area and Fe^{3+}/Fe^{2+} with active growth directions. The espoused surfaces lead to formation of heterogeneous and variable crystal structures. The geodetically-shaped structures could be referred confidently to the kinetic rate of growth reaction, which is strongly tailored by using different Li-precursor agents and their reducing power strength.

In this time-dependent chemical-controlled cathode synthesis, the reducing power of precursor agent is following this order; Li-carbonate > Li-fluoride> Li-chloride, leading to control the growth and complexity degree of large-scale meso-geodesics of (i) conjugated hollow spheres (CHS), (ii) hairy coconut sphere (HCS), and (iii) artichoke flower hollows (AF), respectively. The lower reducing power of Li precursors would probably provide enough time for well development layer-by-layer growth of AF particles with numerous of mesoporous grooves, caves and nests along geode surfaces. Moreover, suitable growth time for hierarchical building of artichoke-flower with full-open-geodes model AF cathode materials also allowed the LFPO atomic arrangement in single crystal building (i.e., orthorhombic olivine structure). A cluster of meso-geodesic complexity deformations eventually plays a subordinate role in creation of low-energy surface functions as transport systems. Multi-functional surface interfaces with long-range of fore- and hind-serrated cuticles and V-undulated ridges, conifers and trial/vein edges, and vortices led to support

longstanding structures of LIB patterns. The walled/fenced AF@C meso-geodesic shell exhibited a huge number of pointed-daggers and abundant of V-undulated ridge-gate windows along edge of 3D surface curvature geode-based cathode electrode for

(i) large coverage distance of the electrodes,

(ii) additional interactions and inertial Li ion loads,

(iii) subsequent diffusion hops in reversal, axial and horizontal directions leverage the motivation of the electron dynamic mobility (i.e., electron-ion-motion systems),

(iv) outstanding discharge capacities and vehicle/truck storage loading, and

(v) harmonized development of high energy density LIB design.

On contrary, Li precursors of high potential reducing agents accelerate the growth reaction kinetics, leading to the formation of (i) hairy coconut sphere (HCS) that have lateral undulations of cladding wave-like movement patterns along cuticular folding with stratified feathery skins, and (ii) conjugated hollow spheres (CHS) that have continuously twisted anticline/syncline ridge-shaped folds or arch-like shape movements. These irregularly located vacancies and geodetically-shaped morphology of both HCS and CHS materials may lead to low mobility of loading capacitance. Furthermore, electronic conductivity and cyclic stability characteristics for pristine LFPO cathode and TiO2 anode have been improved by well-ordered and sustainable ~5nm C-shell dressers along its surface, see Figures 1, S2 and S3. The crystal structures of different morphologies (AF@C, HCS@C, and HCS@C) as cathodes, and TiO_2@C porous nano-sink holes-like vortices (PHV@C-anode) hierarchy composites were further analyzed by HR-TEM as shown in Figs. [S2-(g-j) and S3(d-f)]; respectively. The hairy coconut sphere (HCS@C) exhibits evident crystal planes with interatomic spacing of 0.285 nm, identical to {020} plane. The conjugated hollow spheres (CHS@C) shows evidence of crystal planes with interatomic spacing of 0.38 and 0.425 nm, corresponding to {210} and {-101} planes, respectively.

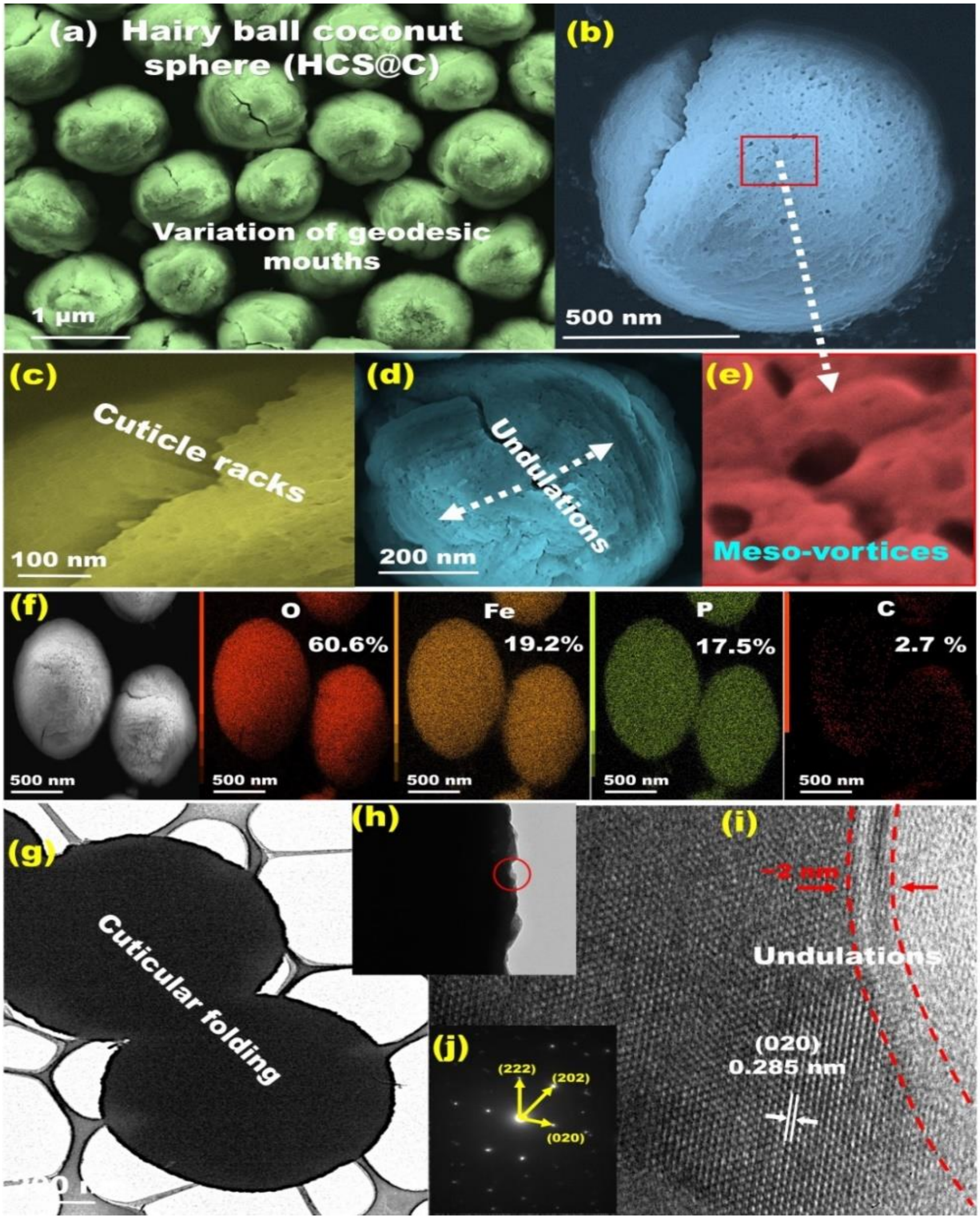

Fig.S2 (a) low magnification and (b, c, d and e) high magnifications FE-SEM of geodetically-shaped hairy coconut sphere HCS, labeled as (HCS@C). (c and d) great cracks extended along surface of sphere. (e) High magnification FE-SEM of diffusive meso-pores, ~10 nm diameter, extended on particle surface. (f) Elemental mapping of HCS @C. (g and h) Low and high magnification HR-TEM micrographs at edge of hand like HCS @C particle. (i) The lattice pattern on surface of HCS @C particle. (j) Selected area electron diffraction (SAED) pattern image of HCS @C unit block indicates the single crystal of HCS @C particles.

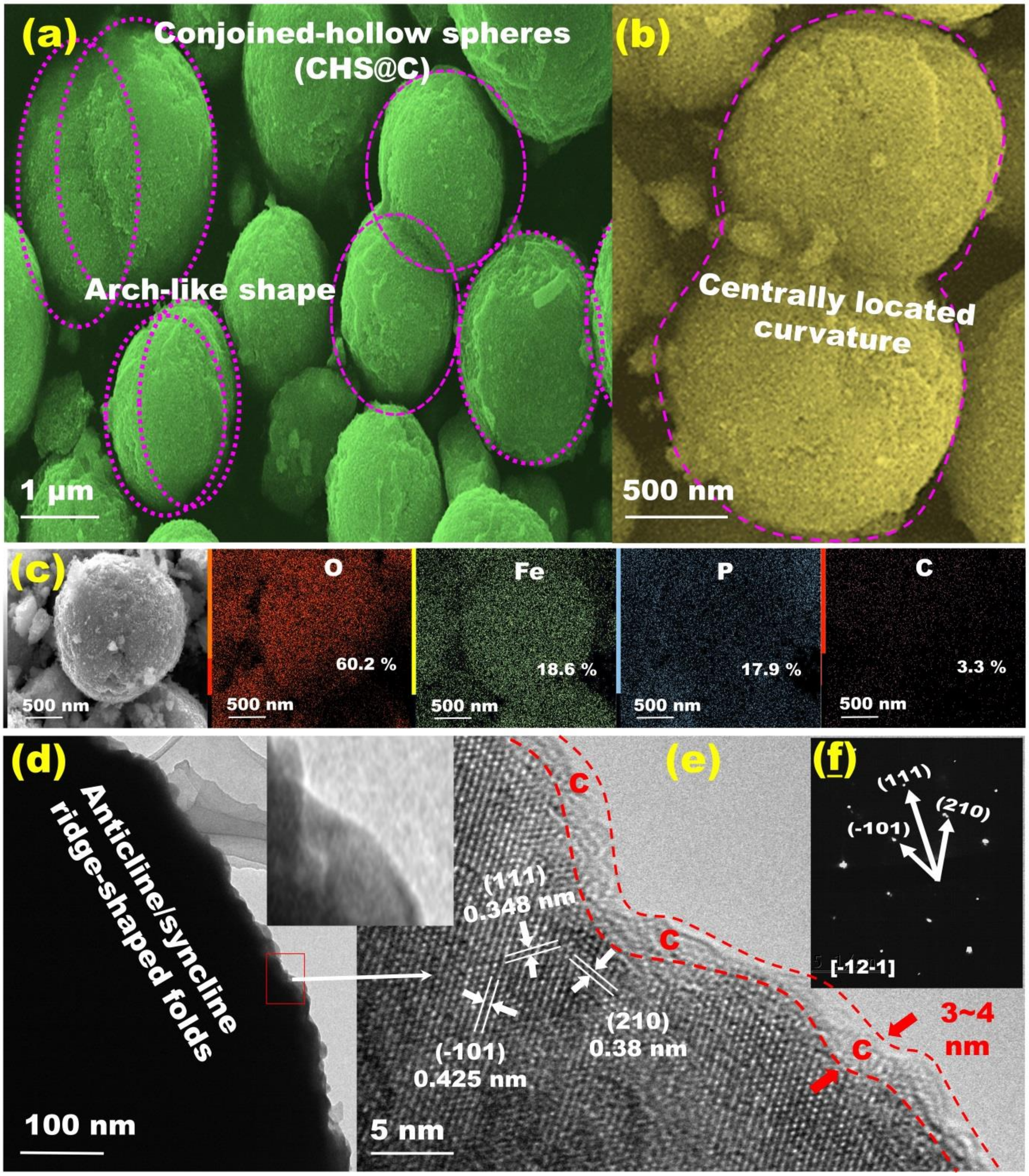

Fig. S3 **(a and b)** Low and high magnifications of FE-SEM for LFPO@C particles with conjugated hollow sphere (CHS), labeled as (CHS@C). **(c)** Elemental mapping of CHS@C. and **(d, e and f)** HR-TEM micrographs with low and high magnifications. **(e)** The lattice pattern at edge with clear thin layer 3-4 nm of C-coating on surface of CHS particle. **(f)** Selected area electron diffraction (SAED) pattern image of CHS@C unit block with incident beam along the [-12-1] crystallographic direction indicates the single crystal of CHS@C particles.

S4. Surface structure parameter and mesoporosity

The textural parameters, dynamic arrangement of space holes, mesocages and pore entrances, and surface coverage area of these scalable architectures provide key broadening effect of the surface morphology on the creation of a mount of diffusion gateways for fast kinetic charge–discharge rates. We measured the N_2 adsorption−desorption isotherms of spheroid-cathodes to determine the specific surface areas (S_{BET} m^2/g) of mesoporous geode structures, as evidence from Brunauer–Emmett–Teller (BET) method, pore size distribution (PSD) curves that determined by using non-linear density functional theory (NLDFT), Fig. S4 (a&b). Our finding indicated that the N_2 isotherms featured a type IV with H_2 hysteresis loop for all tested cathode samples. This finding indicates the formation of mesocage caves and high surface coverages of the entire spheroid-geode structures. The spheroid-geode-type orientation, variable model structures, and geode surfaces are affected the textural parameters, dynamic arrangement of space holes, mesocages and pore entrances, and surface coverage area. For instance, the (S_{BET} m^2/g) value decreases in this order AF@C > HCS@C > CHS@C unit blocks, respectively. Among all geodes structures, the AF design can be considered as diverse surface electrode for excellent specific capacities, facile charge–discharge rates, high-energy-density, and long-timescale stability LIBs. The calculated Brunauer-Emmett-Teller (BET) surface areas are 134.65, 65.07 and 17.72 $m^2.g^{-1}$, and the corresponding pore diameters are 15.58, 60.43; 13.32, 45.00 and 13.31, 46.45 nm for AF@C, HCS@C and CHS@C, respectively.

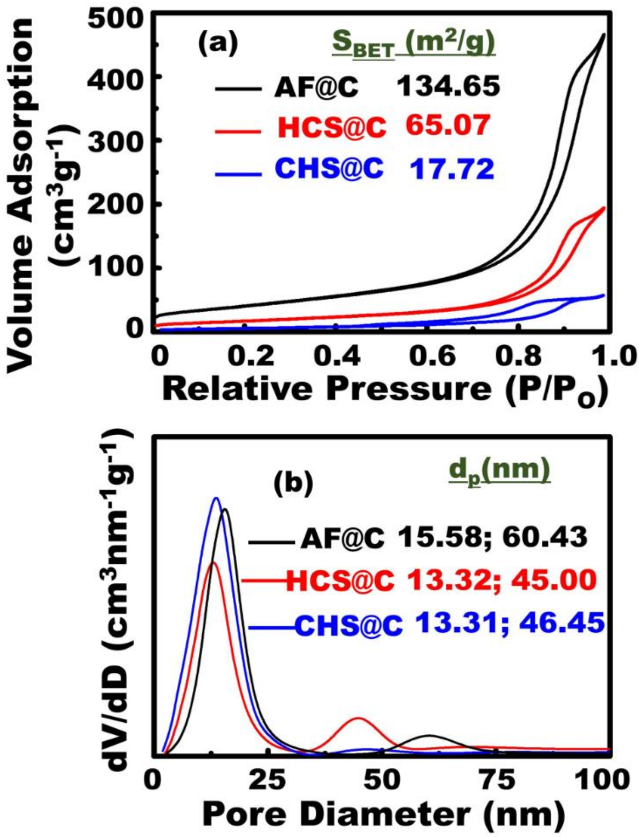

Fig. S4 (a) Nitrogen adsorption-desorption isotherms for AF@C, HCS@C and CHS@C meso-geodesics, including summary of surface areas in m^2/g. **(b)** The corresponding pore-size distribution curves, including summary of pore diameter in nm-scale that calculated by BJH method from the desorption branch for as-prepared AF@C, HCS@C and CHS@C cathode-materials.

S5. Thermal stability of prepared hierarchy cathodes

Thermal stability of prepared samples of geodesic LFPO@C of AF@C, HCS@C, and HCS@C cathode composites clearly observed by and differential scanning calorimetry (TG/DSC) method under a continuous flow of air with a heating rate of 5 °C·min^{-1} as shown in Fig. S5. TG and DSC curves of the A AF@C, HCS@C and CHS@C meso-geodesic cathode composites showed three discrete regions of weight loss or heat transfer at the temperature ranges of 0-1000 ^{o}C. The first insignificant weight loss or heat transfer region below 350°C, is basically produced by releasing of absorbed water and chemisorbed crystal water (desorption of water). The second weight loss or heat

transfer region is from 350°C to 550°C. Significant changes or marked shifts of weight loss or heat transfer of AF@C, HCS@C and CHS@C meso-geodesic cathode composites indicated the endothermic effects. The presence of this endothermic behavior is mainly due to pyrolysis of the non-polymeric organic compounds such as glucose (c-sources) as shell-dressers of AF@C, HCS@C and CHS@C meso-geodesic cathode composites or PHV@ C anodic samples. The third weight loss or heat transfer region above 600°C is evident. No significant change of weight-loss was observed when the temperature increased to 900°C. At >> 600°C, exothermic crystallization peaks are not discovered in DSC curves and the mass of the sample is not changed. This finding indicates that the oxidization reactions of LFPO@C composites were terminated. Therefore, the appropriate calcination temperature is 600°C.

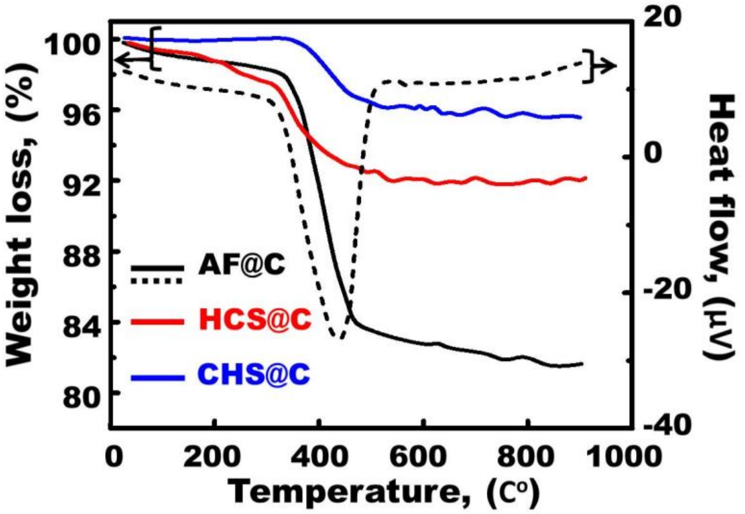

Fig. S5 TG curves of geodesic LFPO@C of different morphologies (AF@C, HCS@C, and HCS@C) as cathode composites. The differential scanning calorimetry, DSC for AF@C can be seen as short-dash curve as example for cathode-material.

S6. Chemical structure of large-scale meso-geodesics

The chemical compositions and framework structure of the AF@C, HCS@C and CHS@C meso-geodesic cathode composites were investigated by Fourier transform infrared spectroscopy (ATR-FTIR, Spectrum 100, Perkin-Elmer, Inc., USA). Fourier transform infrared (FT-IR) spectra of AF@C sample were performed, as shown in Fig. S6. FT-IR spectrum of AF@C exhibits a peak at 578 cm^{-1}, small intense peak around 1440 cm^{-1} and peak at 3250 cm^{-1} that attributed to Fe−O, P−C and $C{=}H_2$ stretching vibrations. Also, strong band centered at 1090 cm^{-1} is ascribed to $-CPO_3$ group tetrahedral stretching vibration. Moreover, the weak peak at 1536 cm^{-1} is due to $C{=}H_2$ bending vibration. Therefore, FT-IR result ensures the present of FeP framework in amorphous pore walls. The C-coated materials featured peaks at 1615 and 1718 cm^{-1} (Fig.S6, star marks), which indicated the aromatic oxides and the existence of C=O stretching.

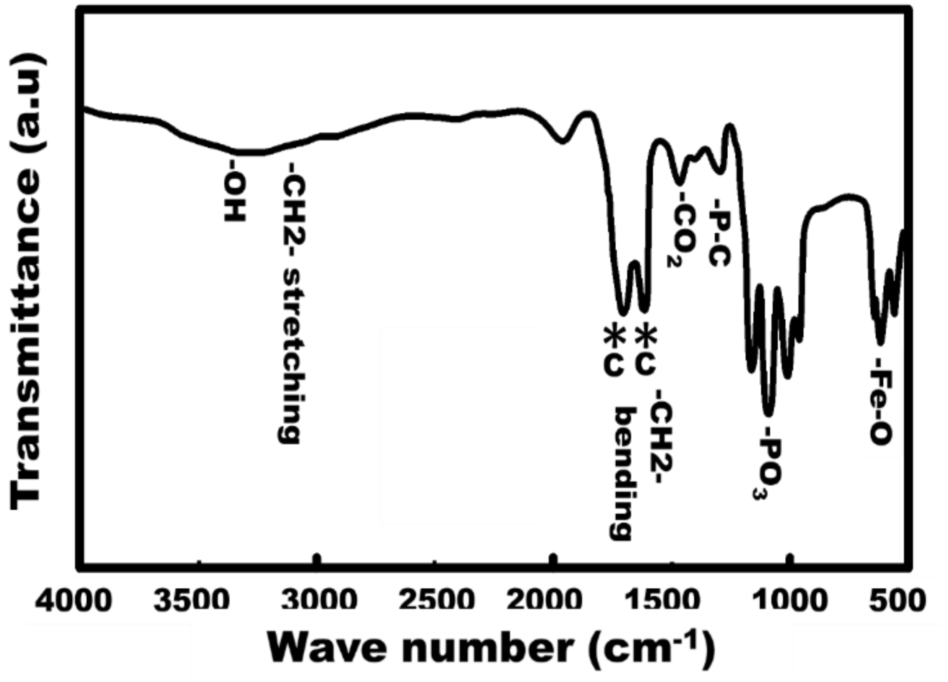

Fig. S6 FT-IR spectra of hierarchical AF@C meso-geodesic cathode composites

S7. Chemical bonding and composition of large-scale meso-geodesics

Raman spectroscopy results for AF@C composite is shown in **Fig.S7.** The bands at 632.0 and 944.3 cm^{-1} agree to symmetric mode of Fe_3O_4 and PO_4^{3-}groups of AF@C. Two peaks at 1337 and 1595 cm^{-1} are attributed to D and G bands of carbon in AF@C, respectively. The C-D-peak ascribed the disordered carbon of highly defective graphite, The C-G- peak is related to (graphite, in-plane vibrations with E_{2g} symmetry). Therefore, according to Raman and FT-IR analyses, the formation of thin carbon layer on surface of AF@C is evident. The analyses also indicated cross-linked binding with pristine LFPO frameworks via C=C, C=N, C=O bonds after carbonization.

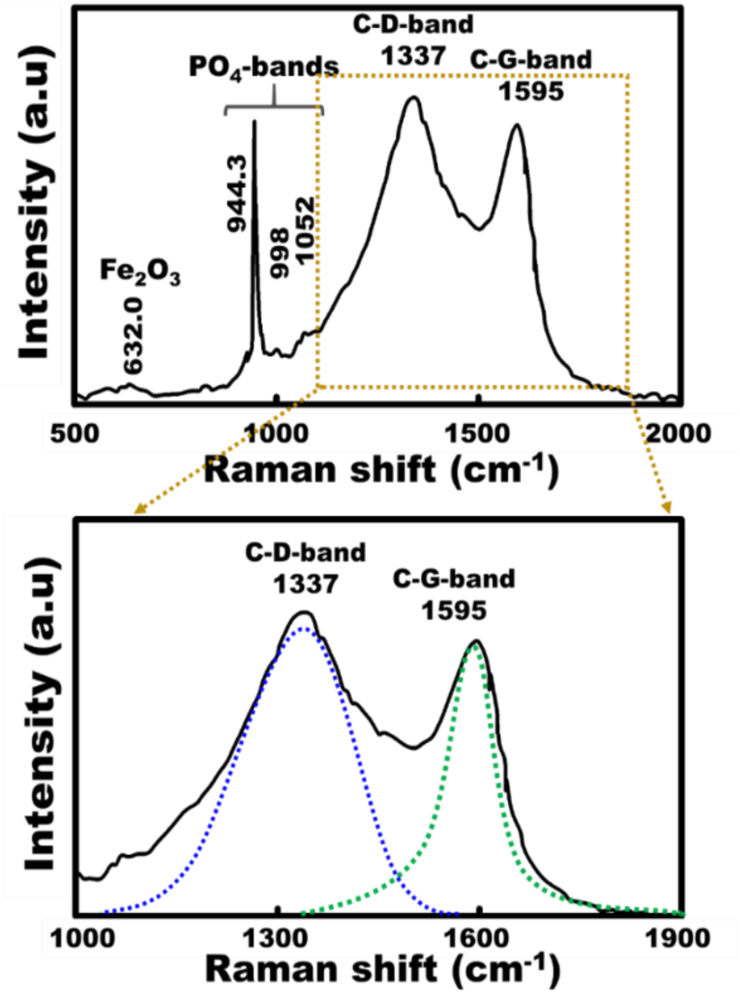

Fig. S7. (a & b) Raman spectra of hierarchical AF@C meso-geodesic cathode composites

S8. Surface binding and chemical composition of large-scale meso-geodesics

XPS spectrum provides chemical surface information on the AF@C sample (Fig. S8). Figure S8 illustrates the XPS spectrum at spectrum peaks at 711.2 and 530.9 eV, indicating the BEs of Fe2p and O1s, respectively. The Fe-BE is related only to its valence state (Fe^{2+}). Some other valence states couldn't observe. P2p, Li1s and C1s peaks are seen at 133.4, eV 55.8 and 284.9 eV, respectively. Then, the XPS results ensured that our AF@C composites are consisted of all elements of olivine LFPO@C, in agreement with XRD profiles.

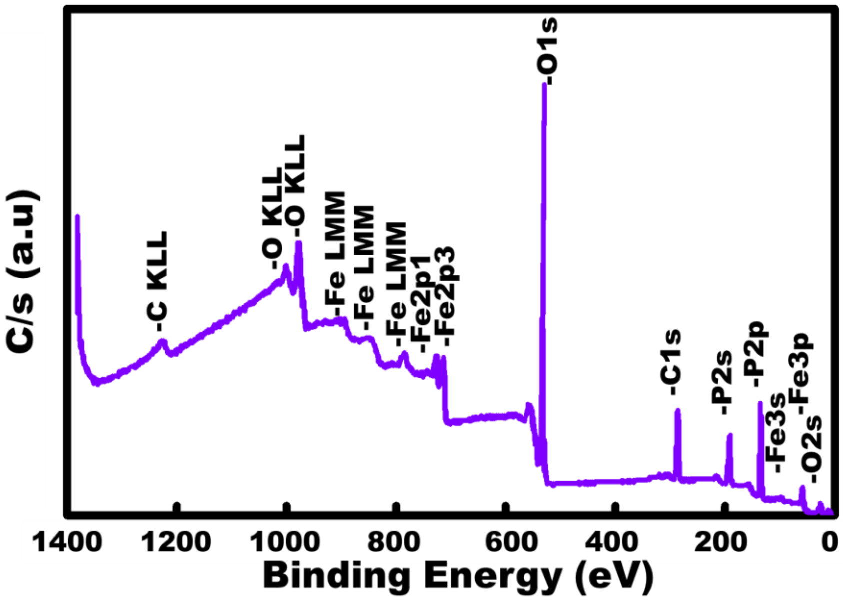

Fig. S8 XPS survey spectrum of the hierarchical AF@C meso-geodesic cathode composites

Together, the XPS, Raman spectroscopy, and FTIR analyses indicated the following:

i. The formation of anisotropic heterogeneous geode composites on both cathode/anode electrodes,

ii. The chemical and physical stability of heterogeneous LFPO@C of different morphologies (AF@C, HCS@C, and HCS@C),

iii. The stability of primary structural tectonics of both cathode (AF@C, HCS@C, and HCS@C), and PHV@C anode, and

iv. Both anode/cathode electrode formulated with multi-functional surface composites, reactively heterogeneous olivine crystal orientation, active surface mobility sites, which are probably responsible for efficient electrochemical performances.

S9. Crystal structure formation of meso/macroporous spheres (PHV@C) anode and LFPO@C cathode hierarchy

We investigate the phase purity and structural properties of geodesic LFPO@C cathode (AF@C, HCS@C, and HCS@C), and PHV@C anode electrodes (Figure S9A&B). XRD profiles show well-deified peaks, which are indexed to the ordering spheroid-geode atomic-scale, and pure orthorhombic olivine-type structure, space group of Pnma (JCPDS card No. 83-2092), Figure S9A. No significant change in no phase structures associated (such as Li_3PO_4, $Li_3Fe_2(PO_4)_3$, or conductive FeP or $Li_4P_2O_7$) with pure $LiFePO_4$ phase is evident. The Rietveld refinement results (a = 9.814 Å, b = 5.817 Å, and c = 4.783 Å, with an acceptable R_{wp} value of 1.238 and unit volume = 286.95Å^3) are all in good agreement with the literature values (JCPDS card No. 83-2092)[27]. $LiFePO_4$-olivine crystal structure with [010] direction and the possible lithium pathways are illustrated as inset of Fig. S9b. The AF@C crystal orientation with high-exposed a-c plane is optimal surface energy for (i) fast electron/ Li^+-ion diffusion dynamics, and (ii) superb kinetics of lithiation and delithiation.

Figure S9B showed diffraction peaks of PHV@C particles. The profile is indexed to tetragonal structure with $I4_1/amd$ space group. The XRD pattern indicated a formation of well-defined pure structure for anatase PHV@C crystal (JCPDS 21-1272), with no observation of phase crystal impurity of rutile and brookite structures. The TiO_2-anatase lattice parameter values were calculated using refinement and found to be a= 3.789 Å, b= 3.789 Å and c = 9.489 Å. This finding provides evidence of the atomic-scale arrangement of the anode designs, which may offer electron/ion

movement regulators along all axes and surface sites. The TiO_2-anatase is suggested as excellent anode material owing to its great merits in safety performance, cheap, non-toxic, pollution-free natures, eco-friendliness, and low polarization. The TiO_2-anatase PHV anode showed good cyclic stability and reversibility, indicating to the structure transition from tetragonal TiO_2 (space group $I4_1$/amd) into orthorhombic lithium-rich $Li_{0.5}TiO_2$ (space group Imma). These PHV@C anodic particles offer multi-directional accommodation sites for facile diffusion of the electrons/Li+-ions diffusion during lithiation/delithiatin processes.

Together, the fast Li^+-ion diffusion with suitable accommodation and a high rate capability for multifunctional surface geodesics anode//cathode can be attributed to the following structural features:

(i) the unique building of 3D-heirerichal meso-geodesics with multi-diffusive connective geodes,

(ii) formation of meso/macro-caves and windows as suitable accommodation sites and enhancing the efficiency of multicentral dimensionality diffusion of Li^+ ions during discharge (insertion, lithiation)/charge(delithiation, extraction) processes, and

(iii) growth of pure crystal orthorhombic olivine LFPO@C cathode and [101]-anatase PHV@C anode responded to short directions for Li^+-ion diffusion.

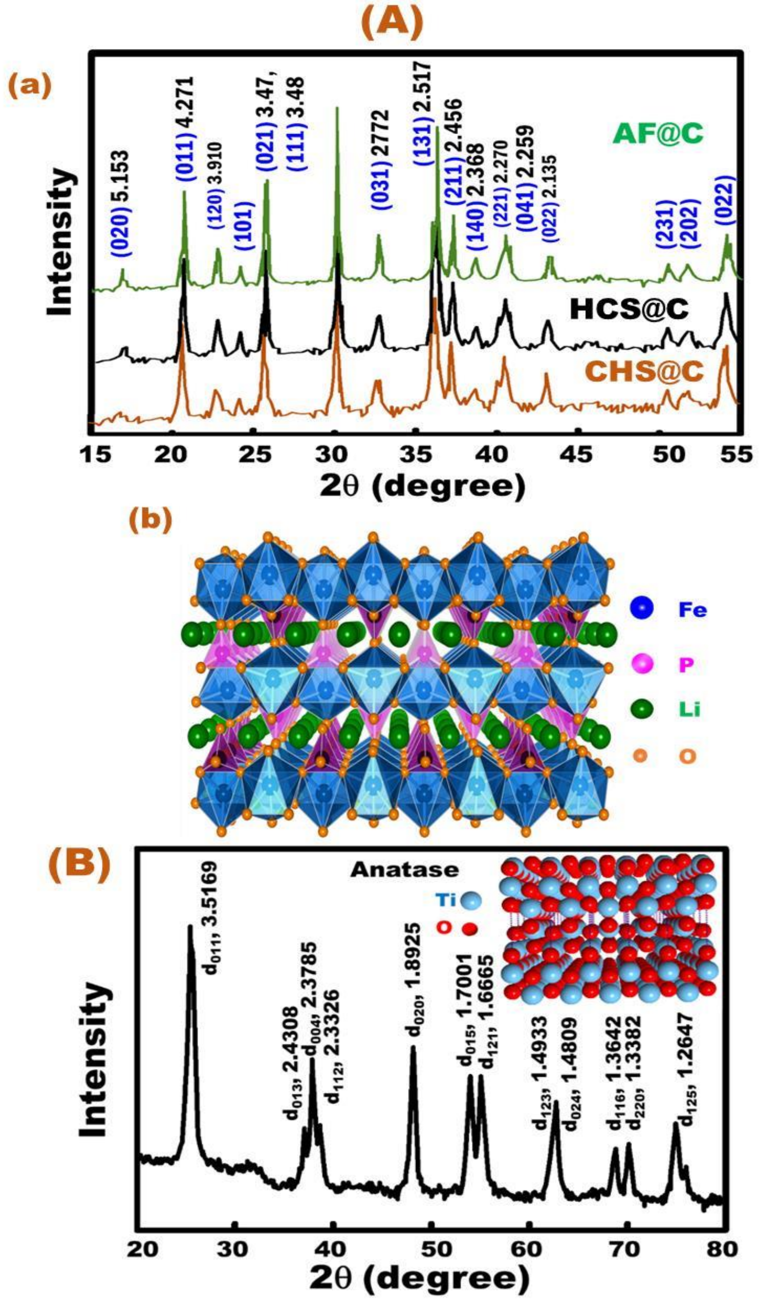

Fig. S9 (A&B) XRD patterns of as prepared geodesic LFPO@C of AF@C, HCS@C, and CHS@C as cathodes (Fig. S9A), and TiO_2@C (PHV@C) anode (Fig.S9B). **(A-b &insert)** Crystal structure of orthorhombic olivine-type structure of [010]-LFPO@C cathode; **(insert)** [101]-anatase TiO_2@C crystal structure of PHV@C anode.

S10. Electrochemical measurements of half-cell meso/macroporous spheres PHV@C anode

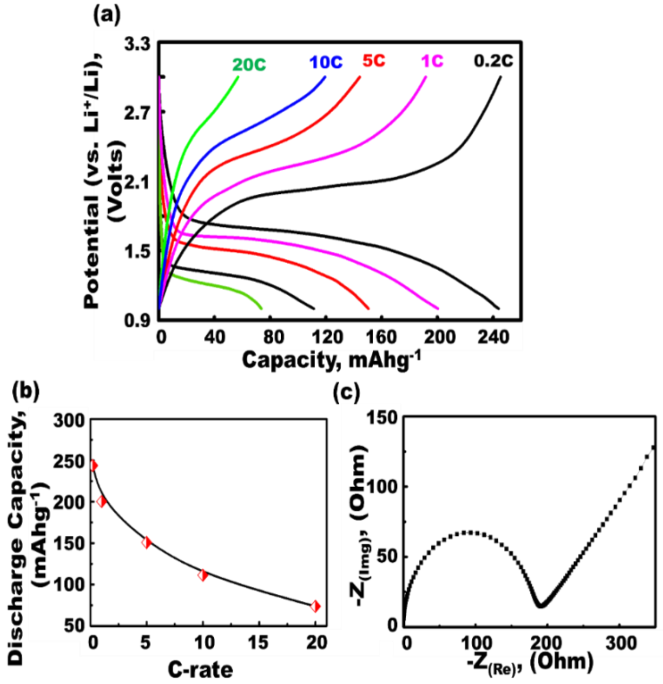

Fig. S10 (a) The charge-discharge voltage profiles of first cycle at multi-current rates 0.2C, 0.5C, 1C, 5C, 10C and 20C for half-cell PHV@C anode material. **(b)** First discharge capacity of all PHV@C at current rates 0.2C -20C of half-cell LIBs. **(c)** The electrochemical impedance spectroscopy (EIS) results of prepared C-coated PHV@C half-cell anode material. All electrochemical measurements for half-cell PHV@C anode materials were operated within voltage range of (1.0-3.0 V), at room temperature.

S11. Rate capability performance of PHV@C meso/macro pores spheres anode

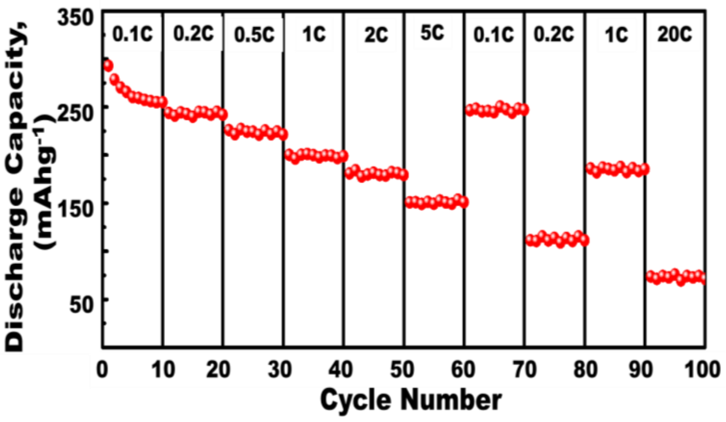

Fig. S11 Rate capability performance rates for half-cell PHV@C anode material at various current rates from 0.1C to 20C. The electrochemical measurements for half-cell PHV@C anode material were operated within voltage range of (1.0-3.0 V), at room temperature.

S12. Calculation of specific energy density of AF@C//PHV@C (LFPO//TiO_2) full-scale LIB model

A. Practical calculation of specific energy density:

In general, the practical specific energy can be calculated during discharge process. It is equal to a definite integral of the discharge voltage (V) over the interval of the discharge capacity, which is ascribed by the obtained voltage versus time (t) profile:

$$\text{Specific Energy} = \int_0^{t_{\text{cut off}}} V(t)\ \text{iAdt} / (3600{*}\text{Mw}) \text{ in Wh.Kg}^{-1}$$

Where: (i) is the current density in Am^{-2}, (A) the area in m^2, (t_{cutoff}) is the time to reach the cut off potential (V_{cutoff}) in seconds, and (M_w) is the molecular weight of the active material used in the electrode.

For galvanostatic cycling test, which was only performed at constant current, the specific energy can be calculated graphically from the charge–discharge voltage capacity profile at 1 C as follows:

Specific energy for the cathode (Wh/kg) = average working voltage of the battery (mid-point voltage) in (V) x Maximum discharge capacity delivered by cell ($Ahkg^{-1}$).

According to the data obtained in Figure 6, the following were concluded:

- The average working voltage of AF@C//PHV@C meso-geodesics full-scale LIB (mid-point voltage in (V) determined from Figure 5) is 1.8 V
- The maximum discharge capacity delivered by cell ($Ahkg^{-1}$) at 1 C in Figure 6 is 165 $Ahkg^{-1}$.

Thus, the specific energy for the cathode (Wh/kg) is 1.8 V x 165 $AhKg^{-1}$, that is, 297 Wh/kg for the cathode.

Based on the mass fraction analysis of cathode in a commercial pouch LIB cell (see Table S1), the specific energy density will be 118.8 $Wh.kg^{-1}$, which was deemed practical for the ($LiFePO_4$//TiO_2) AF@C//PHV@C meso-geodesics full-scale LIB[1].

B. Theoretical calculation of specific energy density:

A suitable combination of negative anode (N) and positive cathode (P) electrode materials leads to a cost-effective and high-capacity LIBs. In our full-scale LIB, LFPO was selected as the positive P-half-cell and anatase TiO_2 as the N-half-cell, respectively.

The nominal open-circuit voltage would be as follows:

$$V_{OC} = V_{+} - V_{-}$$

The nominal voltage equals 3.45 and 1.77 V for the P LFPO cathode and N TiO_2 anode, respectively. These values can be compared with our practical values, as shown in Figs. 5a and 5b and Supporting Information Fig. S10 (a). Thus, the nominal open circuit voltage (V_{OC}) of full-cell was calculated as follows:

$$V_{OC} = 3.45\ V - 1.77\ V = 1.68\ V,$$

where V_{+} and V_{-} are the half-cell potentials of the P and N electrodes, respectively.

The LIB cell capacity is prescribed by the specific capacity ($mAhg^{-1}$) of each electrode material formulated the cell. It is assumed that both P and N electrodes have equal capacities. The theoretical capacity of a LIB cell can be calculated by Faraday's law:

$Q_{theoretical} = (nF) / (3600*Mw)\ mAh\ g^{-1}$

Where n is the number of charge carrier, F is the Faraday constant and Mw is the molecular weight of the active material used in the electrode.

The theoretical capacity Q of a LFPO half-cell is 170 $mAh.g^{-1}$, and similarly for anatase TiO_2 is 335 mAh/g[4] .

With the combination of mass fraction of both P-cathode and N-anode electrodes, we used an approximately ~40% weight allowance for capacity balancing; the electrodes are considered a practical pouch for Li-ion cell components, such as electrolyte, separator, current collectors, and

packaging (Table S1). Thus, the full-scale LIB features a specific capacity of ((170 Ah)/(1 kg+0.51 kg))/1.4, which is about 80.41 Ah kg^{-1}.

The specific energy of the LIB would then be obtained by multiplying the resulting capacity by the expected cell voltage for this combination of electrode materials. Theoretically, the specific energy density of the full-scale $LiFePO_4$//TiO_2 = 1.68 V *80.41 $Ahkg^{-1}$ = 135 $Whkg^{-1}$.

In general, the practical specific energy capacity is lower compared with the theoretical values because all the Li^+ cannot be removed from the lattice site of the active host material-based electrodes. The remaining Li^+ can be removed on the basis of values above the cutoff potential, leading to non-accessible sites. This specific energy density of AF@C//PHV@C meso-geodesic full-scale LIBs exceeded the salutary limits shown through the driving range requirement for EVs. In terms of the material costs and synthesis complexity, the proposed AF@C//PHV@C meso-geodesic full-scale battery system is practical and promising in energy storage applications that needed high energy and power and long cycle life.

S13. Estimation of volumetric energy density

A. Stacked-layer pouch LIB model:

Within the 3D super-scalable full-scale $LiFePO_4$//TiO_2 cathode//anode stacked-layer pouch LIB model, the volumetric energy density can be calculated and found to be 161 Wh/L, according to the following equation:

Volumetric energy density= gravimetric cell energy (118.8 Wh/kg)* total cell mass (0.006 kg)/ cell active area volume (0.004427L) = 161 Wh/L

B. 18650 cylindrical LIB model:

In the test of our proposed $LiFePO_4$//TiO_2 full cell as 18650 cylindrical cells, our proposed LIB

design offers a ~40% mass fraction of the cathode material, whole cell mass of ~0.048 kg, and cell volume of ~0.016 L, then the stored energy density = 118.8 Wh/kg * 0.043 kg = 5.11 Wh, and its volumetric energy density = 5.11/0.016 ~ 320 Wh/L, respectively.

This volumetric energy density of 18650 cylindrical LIB model is reasonably specified in our work and lies in the volumetric energy density range for the most commercial LIB market values, such as 250–500 Wh/L for 18650 LIBs, as shown with the Panasonic NCR18650B and NCR18650GA used as EV LIB[5] .

C. Battery specifications of AF@C//PHV@C full-scale LIB cell:

Given the rapid growth of the EV market, high fuel costs and restrictive regulations are related to environmental and government decisions. The introduction of the LIB is of interest in the transport market, especially in the automotive sector of EVs and HEVs. In terms of large-scale production EV requirements, three LIB designs are used in the EV implementation by common companies: (i) cylindrical (18650, 21700) type (this type is used by Tesla, Lucid, and Faraday); (ii) prismatic type (this type is used by BMW and Volkswagen); and (iii) pouch-shaped type, which features a high module flexibility and capacity, This pouch-shaped type is constrained by manufacture cost and compression control. This LIB pouch-shaped type is markedly used by Chevrolet, Nissan, and Renault. Table S2 presented the battery-specifications comparison between the proposed AF@C//PHV@C full-scale LIB-cell with well-known commercial 18650-cylinderical cells (Table S2).

Table S2 Battery-specifications comparison of the designed AF@C//PHV@C full-scale LIB and other well-known commercial cells.

Cell-<br>Type & Model	Nominal voltage (V)	Min voltage (V)	Max voltage (V)	Mass (Kg)	Volume (L)	Stored Energy (Wh)	Specific energy density (Wh/Kg)	Volumetric energy density (Wh/L)	Cycles	Safety
**Commercial AA Portable Power Corp ($LiFePO_4$) LFP18650T – Cell [1]**	3	2.5	3.8	0.036	0.0165	3.9	108.33	236.4	500-1000	Semi
**Commercial Sony US18650G4–$LiCoO_2$) [2]**	3.6	3	4.2	0.048	0.018	7.2	153	395	500	Not safe & toxic
**AF@C//PHV@C full-scale LIB**	**1.8**	**0.8**	**3.5**	**0.043**	**0.016**	**5.12**	**119**	**320**	**~2000-2500**	**Safe & eco-friendly**

[1] http://www.aaportablepower.com/
[2] https://www.maximintegrated.com/en/app-notes/index.mvp/id/4169

D. Calculation of 18650-AF@C//PHV@C full-scale LIB EV pack parameters and configuration

EV-pack is formed from connection of some modules usually in series. The module is designed by connecting number of cells that have the same (or almost) voltages and capacities. The cell connection set in series (S in short) and in parallel (P in short) to achieve the desired operating voltage (i.e., total battery module voltage) and capacity (i.e., total battery module ampere-hour (Ah)), respectively. The LIBs are connected together in a series connection to increase the voltage of the overall module, and in a parallel connection to increase the capacity of the overall module. The proposed number of AF@C//PHV@C full-scale cells is needed to establish a 50 kWh and 180 V full-packed LIB EV. The calculation was established on the consideration that one pack should consist of 10 modules. Table S3 represented the calculation of battery-specifications of module and pack designed for the proposed AF@C//PHV@C full-scale 18650-LIB.

Table S3 *Battery-specifications of Module and Pack designed of proposed AF@C//PHV@C full-scale 18650-LIB.*

Cell Type& Model	One Module				One Pack									
	Cell configuration	***No. of Cell***	***Nominal Voltage (V)***	***Stored Energy (kWh)***	***No. of Modules***	***No. of Cells***	***Cell configuration***	***No. of Cell***	***Nominal Voltage (V)***	***Stored Energy (kWh)***	***(Wh.Kg-1)***	***Pack Mass (Kg)***	***Our C/P (%)***	***Tesla C/P (%)***
**18650-AF@C//PHV@C full-scale LIB**	*10S.98P*	*980*	*18*	*5*	*10*	***9800***	*100S.98P*	*180*	*50*	*80.6*	*620*	*421.4*	***68***	***63***

S14. A comparison between the AF@C//PHV@C full-cell LIB electrode system and the other reported $LiFePO_4$//TiO_2 full cells

Table S4 A comparison between the 3D super-scalable AF@C//PHV@C LIB-model electrode system and the other reported $LiFePO_4$//TiO_2 full cells

Cathode material	Anode material	Nominal Voltage (V)	Cycles	Specific Capacity mA h g^{-1}	Coulombic efficiency	Ref
$LiFePO_4$@N-doped Carbon	$Li_4Ti_5O_{12}$/Rutile-TiO_2	1.8	Not mentioned	90	Not mentioned	Ref S9
$LiFePO_4$	Anatase TiO_2 hollow nanofibers	1.4	retained 88% of its reversible capacity after 300 cycles	103	> 99 %	Ref S10
$LiFePO_4$	anatase/graphene	1.6	700	127	~ 100%	Ref S11
$LiFePO_4$	TiO_2/ROLi–Li titanium dioxide and lithium n-butoxide (ROLi, R = C4H9)	3.4	Good retention after 200 cycles	130	~100%	Ref S12

$LiFePO_4$	Rutile TiO_2	1.8	retained 50% of its reversible capacity after 40 cycles	150	Not mentioned	Ref S13
$LiFePO_4$	spinel Li_4Ti5O12	1.65	Retain 98.9% after 100 cycles	150	~100%	Ref S14
$LiFePO_4$	Anatase TiO_2	1.6	81% of its initial capacity after 300 cycles at 20C	160	Not mentioned	Ref S15
$LiFePO_4$	Graphene Nanoflake	2.3	operate for over 60 charge−discharge cycles at 1C rate with a reversible capacity of 165 mAhg−1,	165	~100%	Ref S16
$LiFePO_4$	spinel $Li_4Ti_5O_{12}$/C	1.8	Retain 98.1% after 400 cycles	167	~100%	Ref S17
$LiFePO_4$@C (AF@C)	TiO_2@C (PHV@C)	1.8	Retains 91.5% after 2000 cycles	165.8	~100%	**Current Work**

References:

1. Golubkov, A. W., Fuchs, D., Wagner, J., Wiltsche, H., Stangl, C., Fauler, G., Voitic, G., Thaler, A. &Hacker, V. Thermal-runaway experiments on consumer Li-ion batteries with metal-oxide and olivin-type cathodes. *RSC Adv*. **4**, 3633-3642 (2014).
2. Liu, S., Yu, J. & Jaroniec, M. Anatase TiO_2 with Dominant High-Energy {001} Facets: Synthesis, Properties, and Applications. *Chem. Mater*. **23,** 4085–4093 (2011).
3. Chen, J.S., Archer, L.A. & Lou, X.W. SnO_2 hollow structures and TiO_2 nanosheets for lithium-ion batteries. *J. Mater. Chem*. **21,** 9912–9924 (2011).
4. Zuniga, L., Agubra, V., Flores, D., Campos, H., Villareal, J. & Alcoutlabi, M. Multichannel hollow structure for improved electrochemical performance of TiO_2/Carbon composite nanofibers as anodes for lithium ion batteries. *J. Alloys Compd*. **686,** 733–743 (2016).
5. https://en.wikipedia.org/wiki/Lithium-ion_battery; https://www.batteryspace.com/prod-specs/NCR18650B.pdf ; https://www.orbtronic.com/content/Datasheet-specs-Sanyo-Panasonic-NCR18650GA-3500mah.pdf

6. https://www.renata.com/
7. http://www.aaportablepower.com/
8. https://www.maximintegrated.com/en/app-notes/index.mvp/id/4169
9. Wang, P., *et al.* Facile Synthesis of Carbon-Coated Spinel $Li_4Ti_5O_{12}$/Rutile-TiO_2 Composites as an Improved Anode Material in Full Lithium-Ion Batteries with $LiFePO_4$@N-Doped Carbon Cathode. *ACS Appl. Mater. Interfaces.* **97,** 6138-6143 (2017).
10. Zhang, X., Aravindan, V., Suresh Kumar, P., Liu, H., Sundaramurthy, J., Ramakrishna, S., & Madhavi, S. Synthesis of TiO_2 hollow nanofibers by co-axial electrospinning and its superior lithium storage capability in full-cell assembly with olivine phosphate, *Nanoscale.* **5,** 5973-5980 (2013).
11. Choi, D., Wang, D., Viswanathan, V.V., Bae, I.T., Wang, W., Nie, Z., Zhang, J.G., Graff, G. L., Liu, J., Yang, Z. & Duong, T. Li-ion batteries from $LiFePO_4$ cathode and anatase/graphene composite anode for stationary energy storage. *Electrochemistry Communications.* **12(3),**378-381 (2010).
12. Nan, Y., Li, S., Li, B. & Yang, S., An artificial TiO_2/lithium n-butoxide hybrid SEI layer with facilitated lithium-ion transportation ability for stable lithium anodes. *Nanoscale.* **11,** 2194-2201 (2019).
13. Hassoun, J., Pfanzelt, M., Kubiak, P., Wohlfahrt-Mehrens, M. & Scrosati, B. An advanced configuration TiO_2/$LiFePO_4$ polymer lithium ion battery. *J. Power Sources.* **217,** 459-463 (2012).
14. Morales, J., Trocoli, R., Franger, S. & Santos-Pena, J. *Electrochim. Acta* **55** 3075e3082 (2010).
15. Guo, Z., Dong, X., Zhou, D., Du, Y., Wang, Y. & Xia, Y.TiO_2 nanofiber bundles as a high performance anode for a Li-ion battery. *RSC Adv.* **3,** 3352-3358 (2013).
16. Hassoun, J., Bonaccorso, F., Agostini, M., Angelucci, M., Betti, M.G., Cingolani, R., Gemmi, M., Mariani, C., Panero, S., Pellegrini, V. & Scrosati, B. An Advanced Lithium-Ion

Battery Based on a Graphene Anode and a Lithium Iron Phosphate Cathode. *Nano Lett.* **148,** 4901-4906 (2014).

17. Yang, C-C., Hu, H-C., Lin, S.J. & Chien,W-C. Electrochemical performance of V-doped spinel $Li_4Ti_5O_{12}$/C composite anode in Li-half and $Li_4Ti_5O_{12}$/$LiFePO_4$-full cell. *J. Power Sources*. **258,** 424-433, (2014).
